# Reset Controller Design Based on Error Minimization for a Lane Change Maneuver

**DOI:** 10.3390/s18072204

**Published:** 2018-07-09

**Authors:** Miguel Cerdeira, Pablo Falcón, Emma Delgado, Antonio Barreiro

**Affiliations:** Department of Systems Engineering and Automation, School of Industrial Engineering, University of Vigo, 36310 Vigo, Spain; pfalcon@uvigo.es (P.F.); emmad@uvigo.es (E.D.); abarreiro@uvigo.es (A.B.)

**Keywords:** reset control, lane change maneuver, CarSim, ISE minimization

## Abstract

An intelligent vehicle must face a wide variety of situations ranging from safe and comfortable to more aggressive ones. Smooth maneuvers are adequately addressed by means of linear control, whereas more aggressive maneuvers are tackled by nonlinear techniques. Likewise, there exist intermediate scenarios where the required responses are smooth but constrained in some way (rise time, settling time, overshoot). Due to the existence of the fundamental linear limitations, which impose restrictions on the attainable time-domain and frequency-domain performance, linear systems cannot provide smoothness while operating in compliance with the previous restrictions. For this reason, this article aims to explore the effects of reset control on the alleviation of these limitations for a lane change maneuver under a set of demanding design conditions to guarantee a suitable ride quality and a swift response. To this end, several reset strategies are considered, determining the best reset condition to apply as well as the magnitude thereto. Concerning the reset condition that triggers the reset action, three strategies are considered: zero crossing of the controller input, fixed reset band and variable reset band. As far as the magnitude of the reset action is concerned, a full-reset technique is compared to a Lyapunov-based error minimization method to calculate the optimal reset percentage. The base linear controller subject to the reset action is searched via genetic algorithms. The proposed controllers are validated by means of CarSim.

## 1. Introduction

Advancements within the autonomous driving field lead to improvements in many aspects of our lifestyles related to transport systems such as road safety, traffic congestion, transit efficiency and reduction of fuel consumption. As far as road safety is concerned, distracted driving, speeding and drowsy driving are among the leading causes of accident rate. According to the World Health Organization (WHO), road injuries constitute one of the main global causes of death. Concerned with this disastrous occurrence, autonomous vehicles must be endowed with an extensive set of capabilities to provide absolute functionality in the face of the wide variety of situations they confront. In this way, human errors can be minimized, reducing as a result the motor vehicle fatality rate. According to some forecasts, the ever-growing independence, accuracy and effectiveness of autonomous vehicles will lead to, by the end of this decade, limited availability of automated driving functions. It is also expected that, by 2040, autonomous vehicles will be endowed with a broad variety of highly automated functions [1]. Among those functions, there are many which have already been implemented, and are continuously being enhanced, such as Pedestrian Detection (PD), Automatic Cruise Control (ACC), Lane Departure Warning (LDW), Lane Keeping Assist (LKA) and Lane Change Assist (LCA), to name a few ones. All of these functions are part of more complex systems that are closely linked and must work together.

Two of these automated driving functions, lane keeping and lane changing, have been thoroughly studied due to its paramount importance for a self-driving intelligent vehicle, as it is evinced by the numerous articles existing in the literature. In addition to being fully operational for critical situations where safety is at risk, autonomous vehicles must be able to move in compliance with a set of comfort requirements. A great variety of control techniques are employed to that end. For example, in [2], a Model Predictive Control (MPC) approach is employed for controlling an active front steering system in an autonomous vehicle. Ni et al. [3] also explore the use of MPC techniques to solve the problem of autonomously driving a vehicle along a desired path on highway scenarios. Likewise, Jalalmaab et al. [4] employ MPC for highway path planning with time-varying safety constraints and a collision avoidance system. MPC exhibits an excellent performance in lateral control, especially, for aggressive maneuvers where actuator constraints concerning the physical limits (amplitude and slew rate limits) increase in importance [5]. To the contrary, when faced with a smooth maneuver, MPC does not achieve its full potential and behaves like a linear controller. In fact, for linear plants and quadratic cost functions (optimized without the need of reaching the limits), the resulting MPC control is linear. A different control approach is investigated in [6] where an overtaking system for autonomous vehicles equipped with path-tracking and lane-change capabilities is implemented by means of fuzzy control. In [7], an automated lane-keeping system is presented. A fuzzy gain scheduling is employed to tune the steering controller. In [8], the authors provide an analytic approach for the systematic development of sliding mode controllers (SMC) that produce a smooth lane change suitable for use in an Automated Highway System. Imine et al. [9] develop an active steering assistance system for heavy vehicles to prevent lane departure. The control approach is based on a sliding-mode observer and the super-twisting algorithm. Hahn et al. [10] introduce a new two-degrees-of-freedom control structure consisting of a linear controller and a nonlinear model based disturbance compensation for an evasive preventive pedestrian protection system. Wang et al. [11] present a robust output–feedback vehicle lateral motion control strategy considering network-induced delay and tire force saturation. Linear techniques are also employed to tackle lateral control. For instance, in [12], a lane keeping system consisting of a PI controller is combined with the use of active disturbance rejection control (ADRC) to guarantee robustness against vehicle uncertainties and external disturbances. Son et al. [13] present a linear quadratic state feedback regulator for a lane-keeping control strategy with predictive virtual lanes. Authors in [14] develop a nested PID steering control method to perform lane keeping by regulating yaw rate and the lateral offset errors. Likewise, Guldner et al. [15] design a PD steering compensator integrated with a second order lead-compensator. Taylor et al. [16] study several linear regulators for lane keeping: a lead-lag control law, full-state linear controller and an input–output linearizing law. In [17], the authors provide a review of different control techniques for lateral motion control. Namely, $H_{\infty }$, adaptive, fuzzy and PID control. From the technical literature, it can be concluded that linear approaches are convenient for smooth maneuvers where the main magnitudes of the vehicle do not take extreme values. Chapter 4 of monograph [18] contains an exhaustive review of methods for lane keeping and lane change maneuvers, including both linear and nonlinear methods, as well as an extensive list of bibliographic references.

Although there is a wide diversity of techniques for lateral motion control, the variety of situations to which the vehicle is confronted is also very large, and ranges from safe and comfortable situations to intermediate and highly risky maneuvers. The objective of this paper is to study reset control techniques, not merely because they have not been previously applied but mainly because it is believed that there exists a missing gap in the current literature. Smooth maneuvers are satisfactorily addressed by linear control, whereas extreme maneuvers are tackled by special nonlinear techniques (MPC, SMC, etc.). However, there are intermediate scenarios where the required responses are smooth, but constrained in some way (rise time, settling time, overshoot). Due to the smoothness required for those intermediate scenarios, linear techniques are good candidates. The key point concerning them is the existence of fundamental linear limitations (particular restrictions on the achievable time-domain and frequency-domain performance). These limitations could be alleviated by specially targeted techniques (reset control) without the need of employing methods (MPC, SMC) better suited for more aggressive, constrained or uncertain scenarios.

A reset controller is merely a conventional regulator endowed with a reset mechanism which is a strategy that resets to zero (or to a certain percentage) one or several of the controller states, provided that a certain condition is met. The event that triggers the resetting action is usually the zero-crossing of the controller input, although other choices are possible as well. The first existent record in the state of the art concerning reset control is included in the influential work of J. Clegg published in 1958 [19]. In this article, Clegg demonstrated the advantages of reset control compared to linear control and developed what is known as Clegg Integrator (CI). This study was motivated by the following issue. An integrator can be considered to have two delays. The first of them is the time required for the output to reach a certain setpoint after an input signal is applied. The second delay is the time required for the system to get to zero once the input signal goes to zero. This delay does not serve any useful purpose and, in fact, its effect is destabilizing. If the output of the integrator could be taken to zero whenever the input goes to zero, the stability of the system would be improved. This can be achieved by resetting to zero the integrator once its input goes to zero. This is exactly what a Clegg’s Integrator does. In this way, the integrator is reversed to zero immediately after the desired output is achieved. In spite of its advantages, Clegg’s contribution went unnoticed until the early 1970s, when its study was tackled by Horowitz’s research group [20,21]. In these articles, Horowitz highlighted how reset control helps to overcome the well-known fundamental limitations, which affect linear systems [22,23]. After Horowitz’s research was published, the study of reset control was once again discontinued until the late 1990s. Thereafter, the number of research groups interested in this type of control has proliferated significantly.

A remarkable recent proposal is the PI + CI [24], which combines the benefits of a PI controller with those provided by the use of a Clegg Integrator. In recent years, several authors have analyzed the behavior of systems with different reset strategies. One of the first variations studied was the addition of a fixed reset band. This strategy is comprised of a reset mechanism that resets the state(s) of the controller whenever the error signal enters a fixed band. This technique results in being especially beneficial in systems with time delays [25]. The main drawback with a fixed band is that the controller is especially designed for a particular reference (or disturbance). The use of a variable reset band is also studied, this being used for overcoming the influence of dominant time delays over the reset action as reported in [26,27]. A comprehensive monograph on reset control can be found in [28] where different kinds of applications and techniques are included.

As mentioned above, linear systems are affected by what are known as the fundamental limitations. On the contrary, reset systems are known to be unaffected by these restrictions. Considering that tuning a linear controller is relatively straightforward and that linear regulators are especially appropriate for smooth maneuvers, endowing a linear regulator with extra capabilities may be a good alternative to take advantage of the simplicity of a linear regulator while improving its design by incorporating a reset mechanism. The objective of this work is to explore the potential of reset control for a lane change maneuver. To that end, different reset strategies are considered in order to demonstrate which of those yields the best results. As far as the magnitude of the reset action is concerned, a Lyapunov-based ISE (integral square error) minimization method, described in [27] and adapted to the vehicle model, is used to calculate the optimal reset percentage. A full-reset technique is also studied. Concerning the reset condition that triggers the reset action, three strategies are considered: Zero crossing of the controller input, fixed reset band and variable reset band. The regulators originated from combining all these strategies are assessed in terms of robustness in the presence of parametric uncertainties and external disturbances, performance and fulfillment of the design requirements. Adequate design specifications are selected to provide a comfortable response that implies appropriate values of acceleration and jerk. One contribution of this paper consists of guaranteeing comfort while performing a fast lane change by means of a special arrangement of the system that allows direct control over the jerk regardless of the controller employed. It must be noted that, in this work, since the study is focused on the possibilities of reset control, a simple straight-road scenario as well as a step-like input are considered for all the controllers studied. The proposed control technique applied in a low level module could be combined with a more sophisticated technique at a higher level module, which manage the trajectory generation.

To verify the advantages of the proposed method, the reset controllers are compared with two other regulators, an LQR (linear-quadratic regulator), tuned by using Bryson’s rule [29] and a composite nonlinear feedback controller (CNF). CNF is a composite nonlinear control technique that is comprised of a linear and a nonlinear control law directly connected without any switching elements. It was first conceived by Lin et al. in [30] for the tracking control of linear systems subject to saturation in the actuation signal. This method leverages the fast responses of systems with small damping ratios and small overshoots for systems with large damping ratios. The general idea of the method consists of first designing a linear control law capable of yielding a closed-loop system with a small damping ratio. The nonlinear feedback law aims to provide an increase in the damping ratio of the closed-loop system as its output approaches the target reference to reduce the overshoot produced by the linear part. The CNF controller used for comparison is adapted from [31], where it is used for path following.

CarSim (Version 2017, Mechanical Simulation Corporation, Ann Arbor, MI, USA) is employed to validate the feasibility of the proposed reset controllers since it is deemed a standard in the automotive industry. CarSim is used for analyzing vehicle dynamics and assessing performance and it is endowed with a large database of vehicles and automotive elements. Due to its highly reliable models, CarSim is widely used as seen in numerous publications [32,33,34].

This article is organized as follows. In Section 2, the model used for the lane change maneuver is introduced. Then, Section 3 contains all the considerations taken into account for the design of the controllers. After that, some validation results are included in Section 4 and, finally, the conclusions are presented in Section 5.

## 2. Dynamic Model

### 2.1. Description of the Model

**Figure 1:**
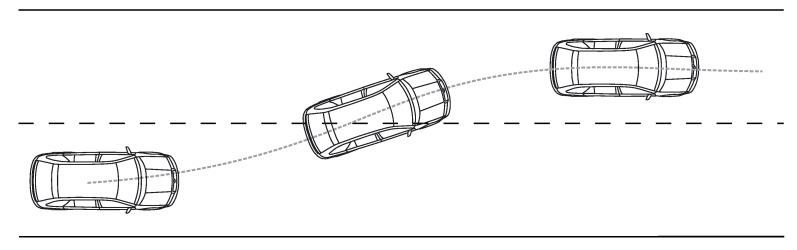
Lane change maneuver.

**Figure 2:**
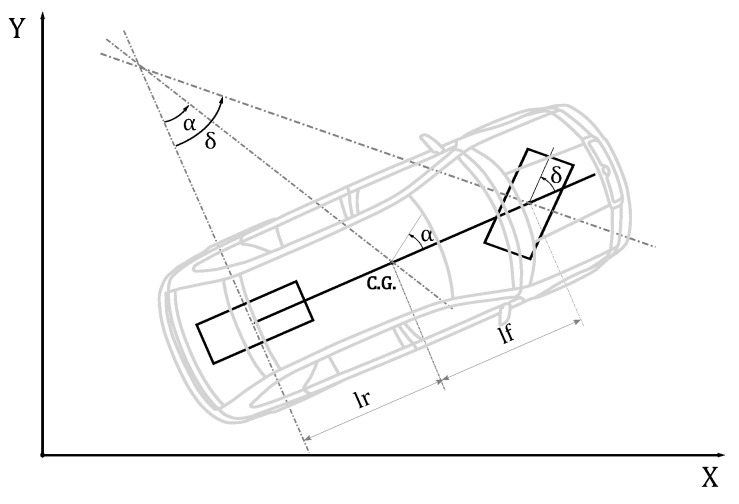
Physical dimensions of the vehicle.

A dynamic bicycle model was employed to model a vehicle before a lane change in a highway. A schematic depiction of the maneuver can be seen in Figure 1. The bicycle denomination has as its source the fact that, as in a bicycle, the model works with the assumption that only two wheels are present, one in the center of each of the two wheel axles. A representation of the bicycle model can be seen in Figure 2.

A detailed description of the whole model and all the intermediate steps and assumptions made to obtain the model can be found in [35]. The parameters included in the resulting linearized model are: vehicle mass (*M*), yaw inertia ($I_{z}$), cornering stiffness of the front wheels ($C_{f}$), cornering stiffness of the rear wheels ($C_{r}$), wheel angle (δ), distance from the front axle to C.G. ($l_{f}$) and distance from the rear axle to C.G. ($l_{r}$). The space-state representation of the model (Equation (1)) can be seen in Equations (2) and (3):

(1) $$\begin{array}{c} \begin{array}{c} \dot{x}\left(t\right)=Ax\left(t\right)+Bu\left(t\right),\\ y(t)=Cx(t)+Du(t), \end{array} \end{array}$$

(2) $$\left[\begin{array}{c} \dot{Y}\\ \dot{\psi }\\ \ddot{Y}\\ \ddot{\psi } \end{array}\right]=\left[\begin{array}{cccc} 0 & 0 & 1 & 0\\ 0 & 0 & 0 & 1\\ 0 & -v_{x}a_{11} & a_{11} & a_{12}\\ 0 & -v_{x}a_{21} & a_{21} & a_{22} \end{array}\right]\left[\begin{array}{c} Y\\ \psi \\ \dot{Y}\\ \dot{\psi } \end{array}\right]+\left[\begin{array}{c} 0\\ 0\\ \frac{C_{f}}{M}\\ \frac{C_{f}l_{f}}{I_{z}} \end{array}\right]\delta ,$$

(3) $$y=\left[1\;0\;0\;0\right]\left[\begin{array}{c} Y\\ \psi \\ \dot{Y}\\ \dot{\psi } \end{array}\right]+\;0,$$

where *Y* and ψ are the lateral position and orientation of the car, respectively. The coefficients $a_{ij}$ are defined as:

(4) $$\begin{array}{cc} a_{11}=-\frac{C_{r}+C_{f}}{v_{x}M} & \;a_{12}=\frac{l_{r}C_{r}-l_{f}C_{f}}{v_{x}M},\\ a_{21}=\frac{l_{r}C_{r}-l_{f}C_{f}}{v_{x}I_{z}} & \;a_{22}=-\frac{l_{r}^{2}C_{r}+l_{f}^{2}C_{f}}{v_{x}I_{z}}. \end{array}$$

This model can be particularized for this case study, with a particular longitudinal speed ($v_{x}$) and the characteristic dimensions of the vehicle, as it can be seen in Table 1. The cornering stiffness coefficient was identified with CarSim. As introduced in Section 1, CarSim is a software considered to be a standard in the automotive industry, used by several manufacturers during the design stage of their vehicles for validation purposes.

**Remark** **1.** *As usual in most vehicle control studies, the justifications for using a linearized model are based on the small-angle approximations (sin(δ)=tan(δ)=δ and cos(δ)=1) in the force and momentum balances in the dynamic model and are also justified by the linear approximation of ground adherence forces, given by the cornering coefficients $C_{f}$ and $C_{r}$.*

### 2.2. Identification of the Model

The vehicle modeled in this work is a Sedan-D Class, a 4-door utility vehicle with 6-speed automatic transmission, 150 kW engine and R17 215/55 tires.

**Figure 3:**
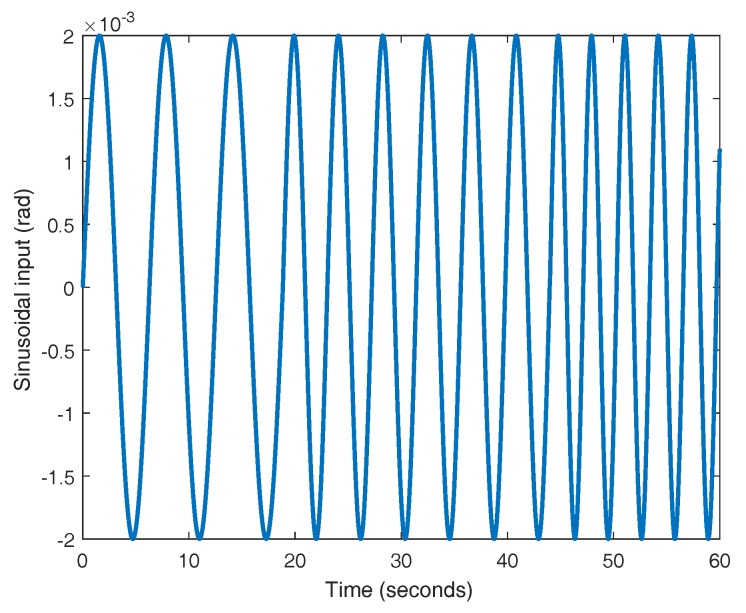
Input signal to CarSim.

In order to get the value for the cornering stiffness coefficient, a well-posed experiment for the identification must be employed. In this case, an open-loop experiment is used. For this plant, the input is the steering angle of both front wheels (δ) and the output corresponds to the lateral position of the center of gravity of the vehicle. To increase the accuracy of the identification, a chirp input signal with a low amplitude is selected (see Figure 3). The test is divided in three time intervals where the frequency of the input signal is different for each interval. Thus, the frequency $w_{i}$ is used for the time interval $t_{i}$, being $\omega_{i}=\left\{1,1.5,2\right\}$ rad/s and $t_{i}=\left\{\left[0,18.75\right),\left[18.75,44\right),\left[44,60\right)\right\}$ s. The amplitude of the signal is in the range of $10^{-3}$ radians to guarantee small angles of δ.

**Figure 4:**
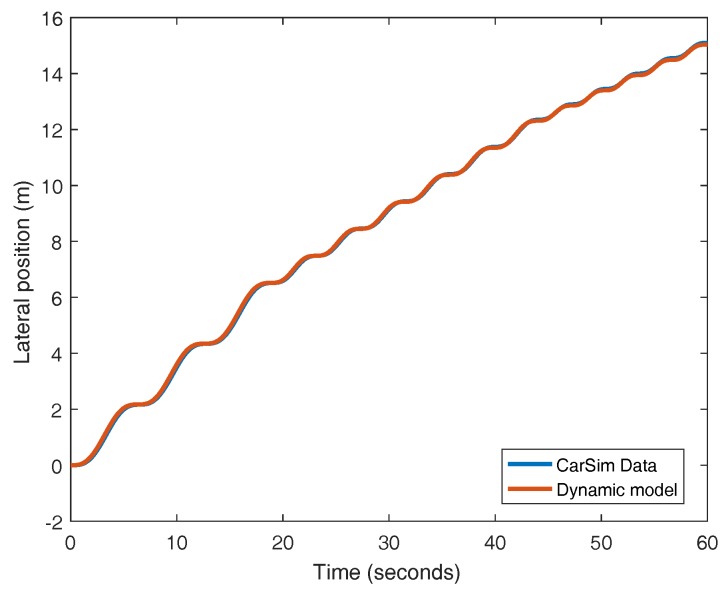
Open-loop responses of the CarSim plant and the identified model.

**Figure 5:**
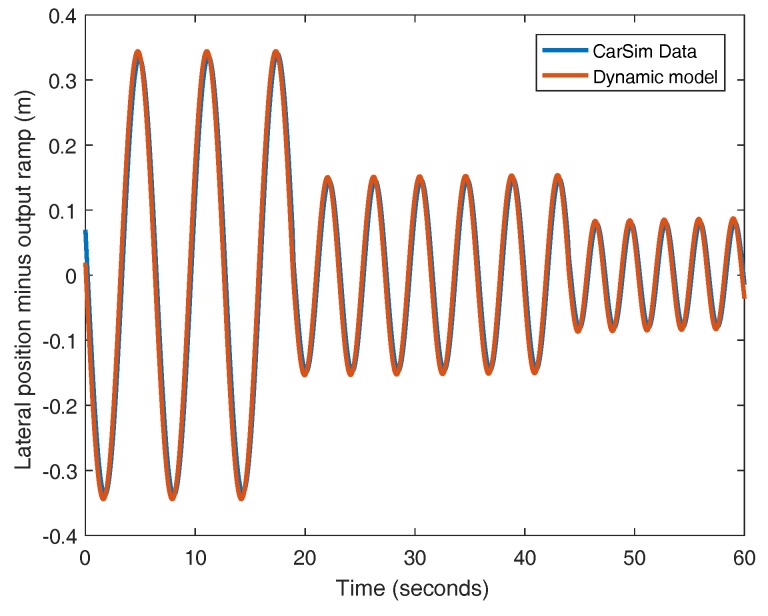
Open-loop responses of the CarSim plant and the identified model without the ramp.

**Figure 6:**
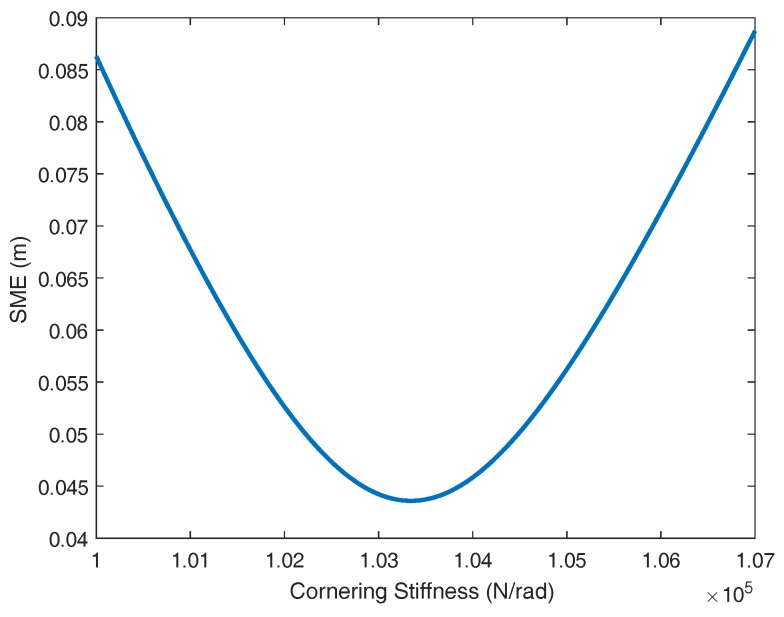
Square mean error between the dynamic model and CarSim data versus cornering stiffness.

In this work, the identification of the cornering stiffness coefficients ($C_{f}$ and $C_{r}$) is performed assuming that both coefficients are equivalent, the tires are equal and the traveling speed is kept constant at 25 m/s. In these conditions, the value of the cornering stiffness obtained is 103,340 N/rad. The responses of both the identified plant and CarSim are very similar, as it can be seen in Figure 4. If the output of the plant is processed and the ramp of the response is removed, the variation of the output signal around zero is obtained (see Figure 5). The difference between the model and CarSim data exhibits a low error for this experiment as depicted in Figure 6.

If the system is represented by its open-loop transfer function for 25 m/s, Equation (5) is obtained. As it can be observed, the plant P(s) has two poles in the origin, and it also has two complex conjugate poles and two complex conjugate zeros:

(5) $$P\left(s\right)=\frac{150.9s^{2}+2501s+3.774\times 10^{4}}{s^{4}+26.43s^{3}+216.5s^{2}}.$$

### 2.3. Model Uncertainty

Uncertainties must be taken into account due to the influence they have over the response of the vehicle since they cannot be included in the model. Examples of these uncertainties are internal parameters of the model that are not perfectly identified or changes in the total weight of the vehicle.

The inaccuracies in the identification process of the model parameters can cause behavioral divergences between the model and the real vehicle. On the other hand, there could be some variability in other parameters such as the cornering stiffness that it is not linear out of a determinate range of slip angle values. This uncertainty can be reduced by performing lane changes where the slip angle is small all the time.

As far as the mass variation of the vehicle is concerned, there may be changes in weight and load distribution on the vehicle, which would result in the center of gravity being displaced and, as a result, variations in the behavior of the vehicle.

**Figure 7:**
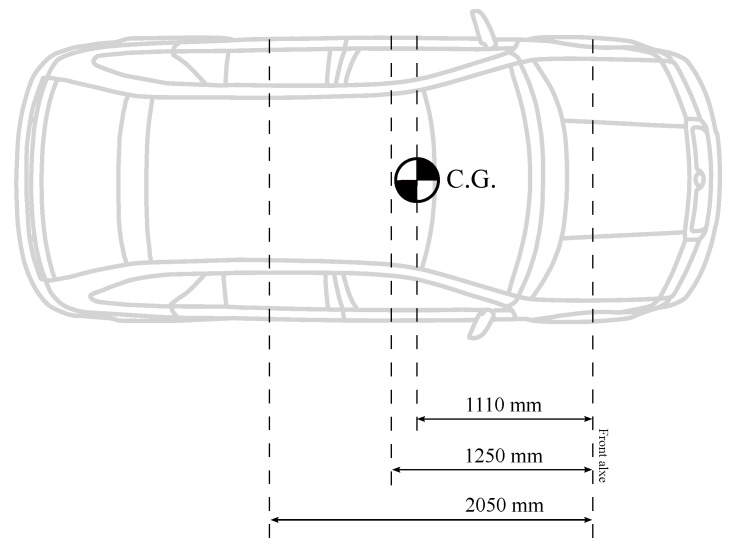
Initial empty car.

**Figure 8:**
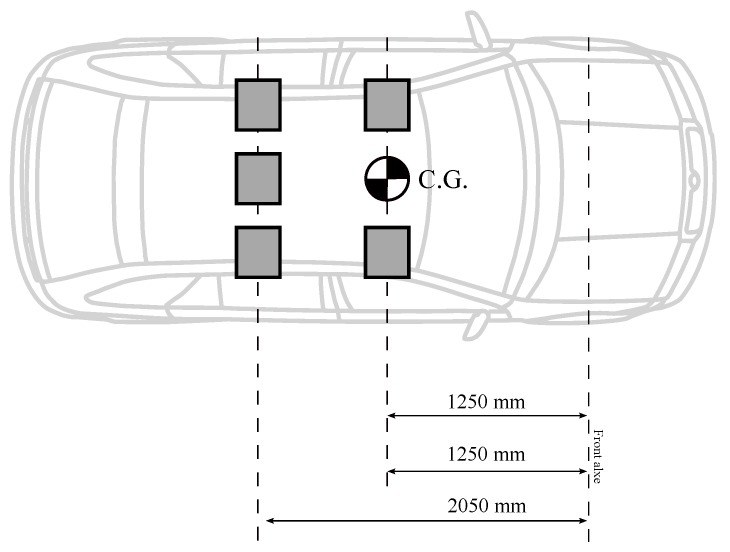
Initial car with payload.

In this work, the vehicle is considered to be fully loaded when it has five passengers with a weight of 80 kg for each of them. These masses cause the movement of the center of gravity towards the rear of the vehicle, so $l_{f}$ and $l_{r}$ are modified. This also results in a change of moment of inertia. In Table 1, the reader can check the difference between the car empty and loaded. Figure 7 and Figure 8 depict how the distribution of masses aboard the car influences the location of the center of gravity.

### 2.4. External Disturbance

Some external conditions to the model are considered in order to evaluate a real situation. In this work, the main external disturbance to take into account is the wind action on the lateral face of the vehicle while it changes from a lane to the other.

**Figure 9:**
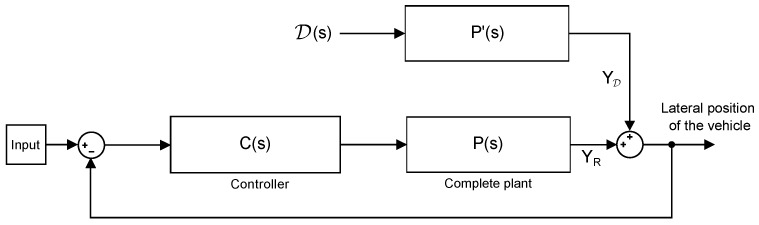
Closed-loop system with disturbance.

To study the system in terms of disturbance rejection, the gain of the system (closed-loop controller and plant) must be calculated when an external force input is applied to the plant. The system is described in Figure 9 where D(t) is the force produced by the wind on the vehicle. In this case, it is assumed that the additional force affects only to $\dot{Y}$ of the state vector and it is perfectly applied in the center of gravity of the vehicle, so it does not produce any momentum. The system used to calculate the incidence of the wind in the trajectory of the vehicle is $P^{\prime }\left(s\right)$, and the system is defined by Equation (1) with a different B matrix, $B={\left[\;0\;0\;1/M\;0\right]}^{⊤}$, which adapts this force input and transforms it into acceleration.

By using the superposition theorem, the total output including the disturbance effect can be seen in Equation (6). For the system in Figure 9 to present a good disturbance rejection, |C(s)|≫1 and |C(s)P(s)|≫1 must hold. In that case, the gain of $Y_{D}\left(s\right)/D\left(s\right)$ is almost zero, and the effect of the disturbance is negligible:

(6) $$Y\left(s\right)=Y_{R}\left(s\right)+Y_{D}\left(s\right).$$

If the action of the wind is a constant force, the system can be studied for low frequencies and then the gain of the closed-loop system faced with disturbance can be calculated with Equation (7). It represents the position deviation in meters for each unit of force in Newtons. Disturbance rejection is considered due to the important effect that it could have on the controlled system, although it can be effectively counteracted by a correct design of the controller C(s):

(7) $$\left|\frac{Y_{D}\left(s\right)}{D(s)}\right|={\left|\frac{P^{\prime }\left(s\right)}{1+C(s)P(s)}\right|}_{s=j\omega =0}.$$

## 3. Control

This section provides a thorough description of all the particulars considered for the adopted control approach. First, Section 3.1 is devoted to the introduction of all the concepts concerning reset control, which is required to understand the development of the control solution proposed for the maneuver at issue. Next, Section 3.2 presents the design requirements considered in order for the end system to operate comfortably. One of these design objectives that involves limiting jerk and acceleration required the vehicle model to be transformed into a double integrator plant by means of a prefilter. Assuming a prefect prefiltering, Section 3.3 focuses on how the base linear controller was obtained and the different reset techniques studied. Section 3.4 covers those details concerning the design of the linear-quadratic regulator as well as the CNF controller for the maneuver under discussion. Finally, in Section 3.5, the outcome of all the controllers is presented and compared.

### 3.1. Reset Control

As mentioned previously, the objective of this work is to explore the potential of reset control for a lane change maneuver. This kind of controller behaves like a linear compensator until the reset action takes place. The reset condition (the event that triggers the reset action) is usually the zero-crossing of the controller input, even though other choices are possible. The linear controller to which the reset mechanism is applied is known as *base linear controller* (BLC):

(8) $$\begin{array}{c} \left\{\begin{array}{c} \begin{array}{c} {\dot{x}}_{r}\left(t\right)=A_{r}x\left(t\right)+B_{r}e\left(t\right),\;if\;e\left(t\right)\neq 0,\\ x_{r}\left(t^{+}\right)=A_{\rho }x\left(t\right),\;if\;e\left(t\right)=0,\\ u\left(t\right)=C_{r}x_{r}\left(t\right)+D_{r}e\left(t\right).\; \end{array} \end{array}\right. \end{array}$$

Equation (8) defines a reset controller with an input e(t) and an output u(t), where x(t) $\in {I\;R}^{n}$ is the state vector, $A_{r}$, $B_{r}$, $C_{r}$ and $D_{r}$ are the system matrices and $A_{\rho }$ is a diagonal matrix whose values vary depending on whether a *full* or *partial reset* is applied. In a *full reset* controller, all of its states are affected by the reset action, whereas, in a *partial reset* compensator, only a subset of them are affected. A variable known as *reset percentage* and denoted as $p_{r}$ is used to adjust the magnitude of the reset action so, for a *full reset* controller, $A_{\rho }$ is a matrix with (1−pr) in all its diagonal elements. On the other hand, for a *partial reset* compensator, $A_{\rho }$ has as many diagonal elements equal to $1-p_{r}$ as there are reset states, having ones in all the diagonal remaining elements. Hereinafter, when the term *full reset* is employed to designate a controller, it will refer to a compensator whose states are fully reset, that is to say, with a $p_{r}$ equal to one, regardless of the number of states affected by the reset mechanism.

The first line in Equation (8) describes the continuous dynamics known as *flow mode*. The second one defines the discrete or impulsive dynamics, known as *jump mode*, due to the fact that, whenever the error crosses zero ($e(t_{k}^{+}=0)$), the controller state jumps from $x(t_{k}^{-})$ to $x\left(t_{k}^{+}\right)=\left(1-p_{r}\right)x\left(t_{k}^{-}\right)$.

As previously mentioned, typically, the condition which triggers the reset mechanism is the zero-crossing of the controller input, and, while it is true that this type of compensator can outperform a well-tuned linear controller, frequently, in control practice, the compensator implementation is done by using a reset band. The use of a reset band may yield better results in terms of stability and performance as noted in [25] for systems with time-delays. Chapter 5 of monograph [28] contains a complete and detailed justification of how a reset band can contribute to enhancing reset control systems.

**Figure 10:**
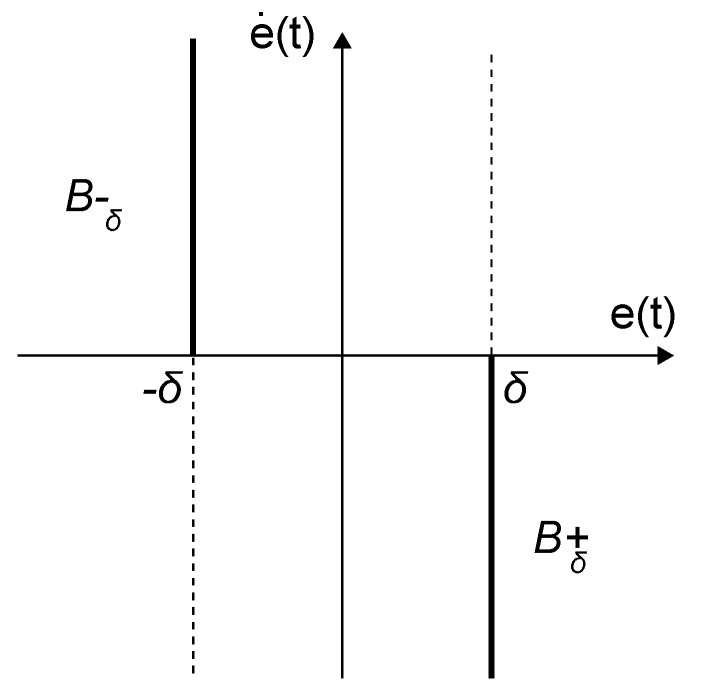
Fixed reset band surface.

A general description of a reset control system with a reset band is given by the following impulsive differential equation:

(9) $$\begin{array}{c} \left\{\begin{array}{c} {\dot{x}}_{r}\left(t\right)=A_{r}x\left(t\right)+B_{r}e\left(t\right)\;\left(e\left(t\right),\dot{e}\left(t\right)\right),\notin B_{\delta },\\ x_{r}\left(t^{+}\right)=A_{\rho }x\left(t\right),\;\left(e\left(t\right),\dot{e}\left(t\right)\right)\in B_{\delta },\\ u\left(t\right)=C_{r}x_{r}\left(t\right)+D_{r}e\left(t\right),\; \end{array}\right. \end{array}$$

where the reset surface $B_{\delta }$ is given by $B_{\delta }=\left\{\left(x,y\right)\in {I\;R}^{2}|\left(x=-\delta \wedge y>0\right)\vee \left(x=\delta \wedge y<0\right)\right\}$, δ being some non-negative real number. In this way, the controller states are reset every time its input enters the reset band. Normally, the reset band surface will consist of two reset lines $B_{\delta }^{+}$ and $B_{\delta }^{-}$, as show in Figure 10. A standard reset compensator is obtained if δ=0.

**Figure 11:**
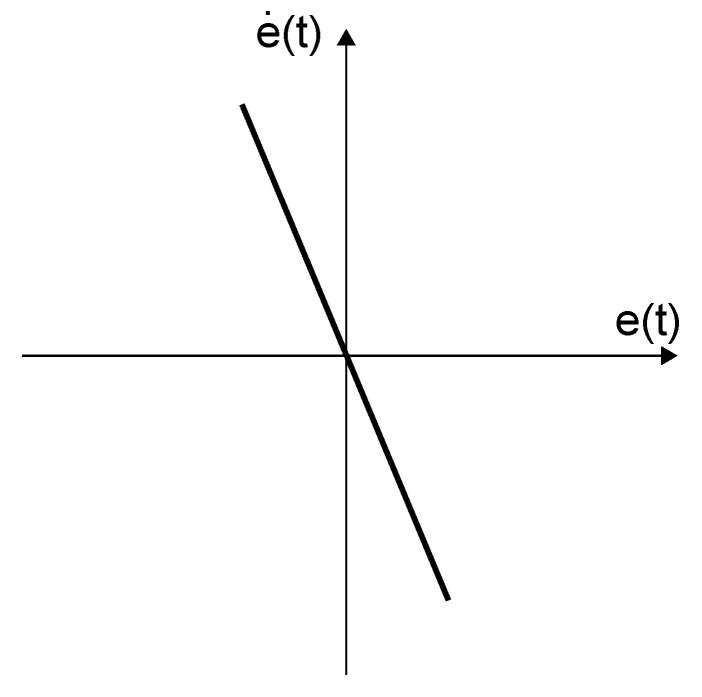
Variable reset band surface.

A variable reset band implies that, at every reset instant, the band value may not be the same. In general, the band value is calculated by a combination of the error and its derivative as seen in the following equation:

(10) $$h\frac{de}{dt}+e\left(t\right)=0,$$

where *h* is a parameter that can be selected at will by the designer. This reset condition leads to the following state-space arrangement of Equation (11):

(11) $$\begin{array}{c} \left\{\begin{array}{c} {\dot{x}}_{r}\left(t\right)=A_{r}x\left(t\right)+B_{r}e\left(t\right),\;\left(e\left(t\right),\dot{e}\left(t\right)\right)\notin B_{h}^{v},\\ x_{r}\left(t^{+}\right)=A_{\rho }x\left(t\right),\;\left(e\left(t\right),\dot{e}\left(t\right)\right)\in B_{h}^{v},\\ u\left(t\right)=C_{r}x_{r}\left(t\right)+D_{r}e\left(t\right),\; \end{array}\right. \end{array}$$

where the variable reset band surface $B_{h}^{v}$ is given by $B_{h}^{v}=\left\{\left(e\left(t\right),\dot{e}\left(t\right)\right)\in {I\;R}^{2}|h\dot{e}\left(t\right)+e\left(t\right)=0\right\}$. The reset surface is a continuous function of the error signal, as it can be seen in Figure 11. If h=0, a standard compensator with reset action triggered by a zero-crossing is obtained.

### 3.2. Design Requirements

Ride quality or ride comfort refers to the feeling that passengers get while the car is moving. Acceleration and its time derivative, jerk, affect ride quality in a prominent manner so that high values of acceleration or jerk can cause discomfort even during short periods of time. For that reason, setting restrictions on magnitude of acceleration and jerk is strictly necessary to guarantee comfort to the vehicle occupants. Limit values vary between different studies, but they fall within the same range. According to [36], the vehicle acceleration must be limited to a maximum value of 2 m/s${}^{2}$ and the jerk to 0.9 m/s${}^{3}$, both in absolute value.

Due to the aforementioned restrictions, the reset instants must be carefully overseen in order not to surpass the limits of acceleration and jerk since, at those particular moments, the jumps in the controller states are critical and may lead to a poor ride quality resulting in discomfort for the passengers. For that reason, during the controller design, a method was conceived to directly restrict the value of jerk every time a reset action occurs. Firstly, for this method to work, it was necessary to convert the dynamic model into a double integrator. On account of this, a prefilter has to be employed for a pole-zero cancellation. It must be noted that, by confining the jerk magnitude to finite values, the resulting acceleration will also be bounded.

The parametric uncertainty that there exists in every vehicle, due to the wide variety of different situations they encounter, makes the use of a single prefilter unfeasible for every possible situation. The way through which the parametric variation affects the model was studied to help to devise the best alternative for prefiltering the plant. Every possible variation of the model parameters was considered to be restricted to the values in Table 2. In order for the vehicle to be fully operational, for any given longitudinal velocity, a prefilter would have to be obtained and included in a lookup table. To reduce the size of this table, instead of having to calculate a prefilter for one particular velocity, each and every one of them will be calculated to be operational for a small interval of velocities ranging 1 m/s. Therefore, if the vehicle accelerates while changing lanes and exceeds its range of operation, the prefilter will be replaced by the most convenient one in terms of velocity. In any case, to exemplify the method, the main lane change maneuver that is considered in Section 4 was chosen to operate at 25 m/s. The longitudinal velocity of the vehicle is considered to be kept constant during the maneuver by a different control loop whose study is out of the scope of this paper.

**Figure 12:**
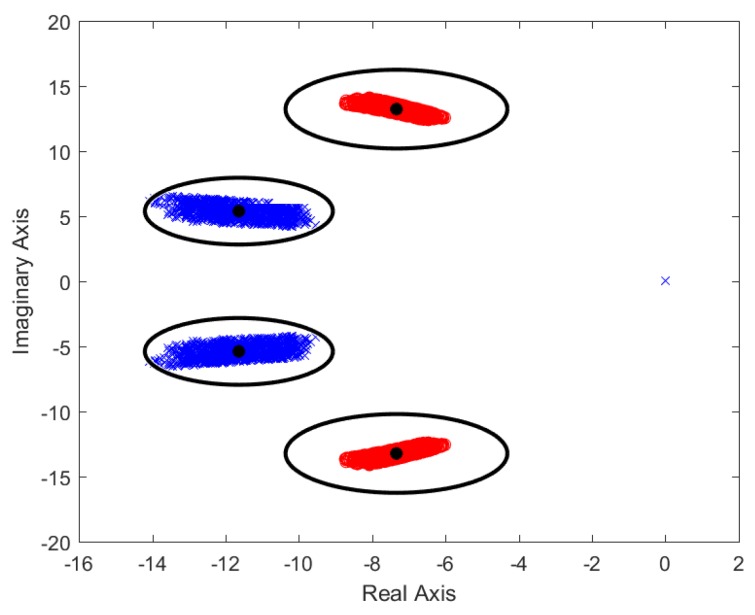
Pole-zero map for a randomly generated parametric sweep of the vehicle model.

A random parametric sweep, using the values from Table 2, was performed to obtain a realistic set of vehicle plants that could be used to obtain a fine prefilter. Since a pole/zero is deemed to be adequately canceled if the zero/pole employed for its elimination is located within a circle with a radius equivalent to the 20% of the total distance existent from the origin of coordinates to the zero/pole, the best way to ensure a good cancellation of all the possible plants is to place the zero/poles of the prefilter at a point equivalent to the arithmetic mean of the cloud of points obtained by the parametric sweep. The gain of the prefilter is obtained through the same way, its value being the inverse of the arithmetic mean of all the different plant gains. As an example, Figure 12 has a depiction of the zero-pole positions (black dots) and the surrounding areas of cancellation for a randomly generated parametric sweep for a longitudinal velocity ranging from 24.5 to 25.5 m/s.

Even though it is true that the core of the validation section is focused on a lane change at a constant velocity, in order to demonstrate the feasibility of the method for a speed changing maneuver, more prefilters are fixed. These are included in Table 3.

**Figure 13:**
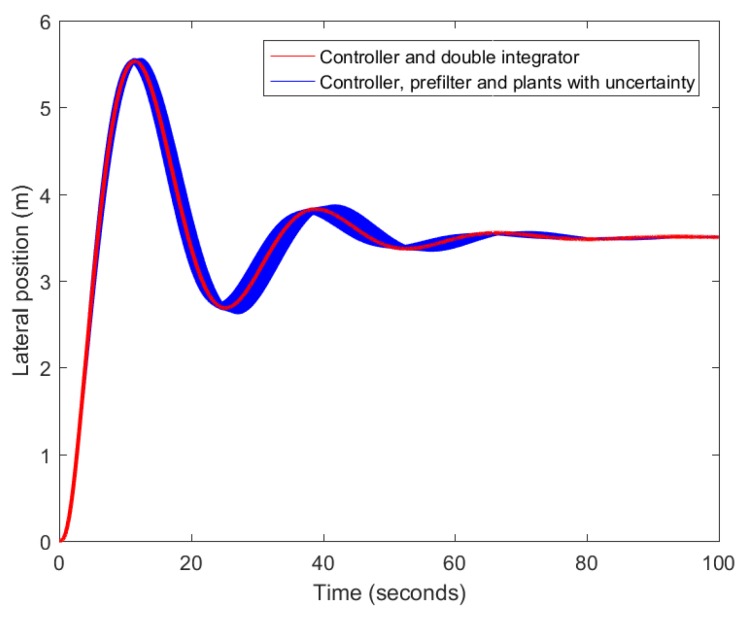
Comparison between the controller loops of the prefiltered and the ideal plant.

**Figure 14:**
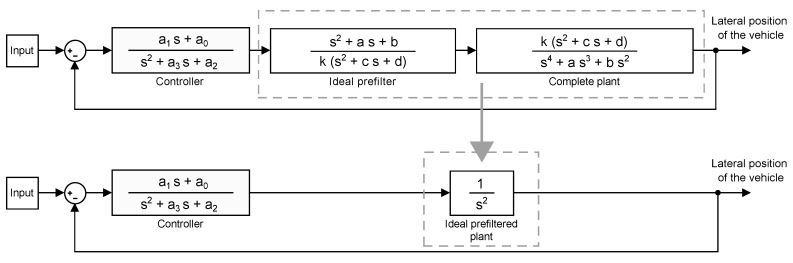
Equivalence of a perfectly prefiltered system.

Using the final base linear controller, with the following realization $\frac{a_{1}s+a_{0}}{s^{2}+a_{3}s+a2}$ and whose design is explained in Section 3.3, the randomly generated plants, prefiltered by using the arithmetic mean of the scattered clouds of zero-poles and gains of the plant, are compared to the system resulting from combining the controller with a double integrator. In this way, the effectiveness of the prefilter can be graphically confirmed in terms of the output of the system, the lateral position of the vehicle. After inspecting the outcome presented in Figure 13, it can be concluded that assuming a zero-pole cancellation for a particular velocity is convenient. Figure 14 depicts the equivalent system of a perfect zero-pole cancellation.

As stated before, by using a double integrator, it is possible to limit the jerk at the reset instants. This is achieved by conveniently reorganizing the states of the resultant system, which consists of a controller and a double integrator. The new distribution of the system states allows for the reset action to be directly applied to the jerk state in order not to exceed the comfort boundary. This new arrangement of the system will be hereinafter referred to as canonical form. Equation (12) shows its state-space representation and it is equivalent to $\dot{X}=AX+Bu$. As it can be seen, the system has been changed to one whose states are the lateral position and its time derivatives ($x_{1}=position$, $x_{2}=velocity$, $x_{3}=acceleration$ and $x_{4}=jerk$):

(12) $$\left[\begin{array}{c} \dot{x_{1}}\\ \dot{x_{2}}\\ \dot{x_{3}}\\ \dot{x_{4}} \end{array}\right]=\left[\begin{array}{cccc} 0 & 1 & 0 & 0\\ 0 & 0 & 1 & 0\\ 0 & 0 & 0 & 1\\ -a_{0} & -a_{1} & -a_{2} & -a_{3} \end{array}\right]\left[\begin{array}{c} x_{1}\\ x_{2}\\ x_{3}\\ x_{4} \end{array}\right]+\left[\begin{array}{c} 0\\ 0\\ 0\\ 1 \end{array}\right]ref.$$

**Figure 15:**
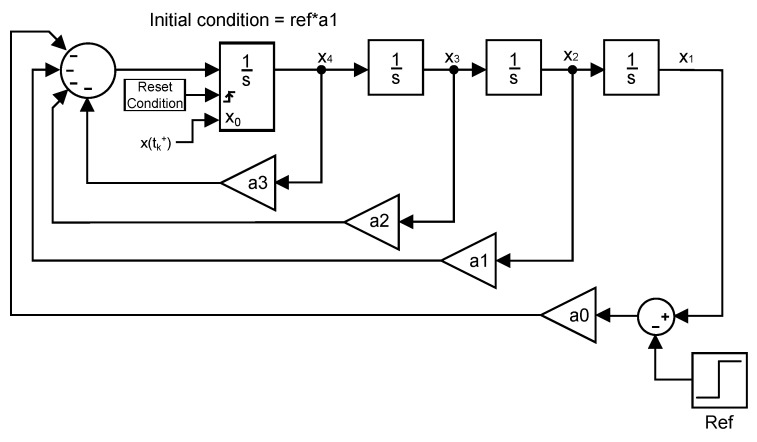
Canonical form.

The fact needs to be taken into consideration that, in order for the canonical form to be equivalent to the system in Figure 14, the initial condition of the reset integrator has to be adapted to obtain a response equal to that of the original system. Figure 15 shows a representation of the canonical form where it can be appreciated that the reset action is directly applied to the jerk state, as reported above.

The comfort limit imposes the maximum variation of the reset state in order to not impoverish ride quality. If after resetting the state, $x_{4}\left(t_{k}^{+}\right)$ exceeds the maximum allowed jerk, $p_{r}$ will be limited as indicated in Equation (13). As far as acceleration is concerned, although it is not directly limited, by obtaining a good base linear controller, with a sufficiently smooth response, the vehicle will operate far from the comfort boundaries. Therefore, whenever a reset action occurs, only jerk but not acceleration will be at risk of exceeding the comfort limits. Likewise, yaw rate is not directly limited due to the fact that the imposed comfort requirements combined with limitations on rise time, settling time and overshoot will confine the values of this magnitude in a safe range of operation. The results in the following sections confirm that this approach gives rise in practice to acceptable ranges of acceleration and yaw rate:

(13) $$\begin{array}{c} \left\{\begin{array}{c} p_{r}=1-\left(0.9/x_{4}\left(t_{k}^{-}\right)\right),\;x_{4}\left(t_{k}^{+}\right)>0.9,\\ p_{r}=1+\left(0.9/x_{4}\left(t_{k}^{-}\right)\right),\;x_{4}\left(t_{k}^{+}\right)<-0.9. \end{array}\right. \end{array}$$

Other design requirements are overshoot, rise time, settling time and disturbance rejection. Lateral position is allowed to reach a maximum equal to the width lane (3.5 m) plus an extra 0.75 m that accounts for an overshoot of 21.45%. Settling time (2%) and rise time have been limited to 40 s and 5 s. The rise time requirement is imposed indirectly by the restrictive acceleration and jerk requirements. It must be noted that both acceleration and jerk requirements tend to make the system slower, whereas the rise time limitation imposes a limit in order for the system not to be too slow. By getting to a compromise between them, the system is fast enough without producing discomfort. $\left|\frac{Y_{D}\left(s\right)}{D(s)}\right|$ is restricted to a maximum of 0.005 m/N all along the frequency domain. Although the controller designed is not intended to work as a lane keeping compensator, guaranteeing a certain degree of disturbance rejection was deemed necessary.

### 3.3. Design of the Reset Controllers

Due to the fact that there are several restrictive design requirements that have to be met, the base linear controller is obtained by means of a genetic algorithm. This is a method for solving optimization problems based on a natural selection process that imitates biological evolution. The ideas involved in them were originally developed by Holland [37].

In general, a typical genetic algorithm may comprise the following elements [38]:

- a population of guesses of the solution to the problem,
- a way of assessing how good or bad the individual solutions within the population are,
- a method for mixing fragments of the better solutions in order to form, on average, better solutions, and
- a mutator operator is employed for the genetic algorithm not to result in a permanent loss of diversity within the solutions.

In the case at issue, each member of the population consists of four parameters or genes which are the coefficients of the controller ($a_{0}$, $a_{1}$, $a_{2}$ and $a_{3}$) as in Equation (14). This controller structure was chosen since it is the simplest realization to which a reset mechanism could be applied without directly resetting the actuation signal, which would produce jumps in the acceleration and, as a result, extreme jerk. For that reason, a second pole was added to the minimum resettable realization:

(14) $$C\left(s\right)=\frac{a_{1}s+a_{0}}{s^{2}+a_{3}s+a_{2}}.$$

In this way, each individual represents a controller. In order to determine the suitability of each one, a fitness function is employed. This expression combines performance information of the system formed by combining each individual controller with the double integrator plant. Acceleration, jerk, overshoot, settling time, rise time and disturbance rejection are included in the fitness function. Each one of them is normalized and then weighted as it is reflected in the following equation:

(15) $$FF_{GA}=w_{1}\frac{max\left|\frac{Y_{D}}{D}\right|}{0.005}+w_{2}\frac{max_{acel}}{2}+w_{3}\frac{max_{jerk}}{0.9}+w_{4}\frac{OS}{21.45}+w_{5}\frac{t_{s}}{40}+w_{6}\frac{t_{r}}{5},$$

where $max_{acel}$ is the maximum magnitude of lateral acceleration, $max_{jerk}$ is the maximum magnitude of lateral jerk, OS overshoot, $t_{r}$ rise time, $t_{s}$ settling time and $max\left|\frac{Y_{D}}{D}\right|$ the maximum gain of the referred transfer function. $w_{i}$ is the weight of each variable, all of them are calculated assuming a perfect pole-zero cancellation so the double integrator plant is used in the genetic algorithm. Therefore, the base linear controller only have to be computed once and it can be used for other velocities due to the homogenizing effect of the prefilter. To compute $max\left|\frac{Y_{D}}{D}\right|$, $P^{\prime }\left(s\right)$ in Equation (7) is calculated for the vehicle without any additional load and traveling at 25 m/s.

The implementation is done by using Matlab Optimization Toolbox and consists of the following steps:

1. A populations of size 50 is randomly initialized within the lower and upper bounds of $a_{0}$, $a_{1}$, $a_{2}$ and $a_{3}$.
2. Each member of the current population is scored by computing its fitness value from Equation (15).
3. Five percent of the individuals with the lowest fitness are chosen as elite and directly pass to the next generation. These are known as *elite children*.
4. Eighty percent of the remaining 95% of the descendant generation is obtained by combining the genes of a pair of parents. These are known as *crossover children*.
5. The rest of the specimens to complete the new generation are created by introducing random changes, or mutations, to a single parent. These are known as *mutation children*.

In each iteration, a different individual of the population is simulated and the parameters of OS, $t_{r}$ and $t_{s}$ are obtained. At the same time, the acceleration and jerk values are calculated as well as the disturbance rejection of the system. Next, all the values are introduced in the fitness function to evaluate the candidate. The computational cost of each iteration obtained has an average of 150 ms and the stopping criterion selected is the function tolerance, whereby the value of fitness function decreases less than $10^{-6}$. In addition, the maximum number of iterations is set to 100 × number of variable = 400.

A base linear controller does not present the best attainable features for a lineal controller, nor is it conceived to have them. OS and $t_{s}$ are assigned a fewer weight ($w_{4}$ and $w_{5}$) in the fitting function since there is room for the reset action to correct the maneuver performance back to operational range. Equation (16) contains the base linear controller found by the genetic algorithm. The system formed by this regulator and the double integrator plant have an overall response that adjusts to the comfort limits, rise time and disturbance rejection. By contrast, $t_{s}$ and OS maximum allowed thresholds are exceeded:

(16) $$C{\left(s\right)}_{BLC}=\frac{0.2571s+0.0683}{s^{2}+1.8379s+1.4872}.$$

**Figure 16:**
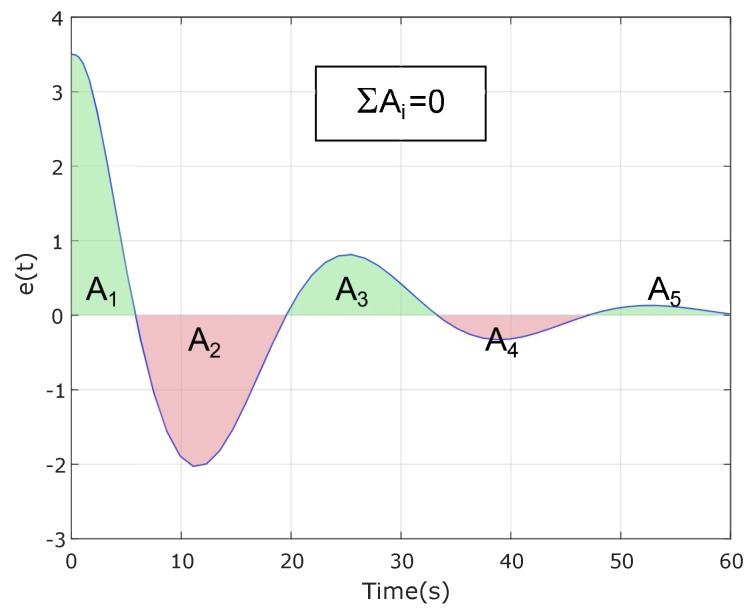
Error of a system formed by a linear controller with a double integrator plant.

**Figure 17:**
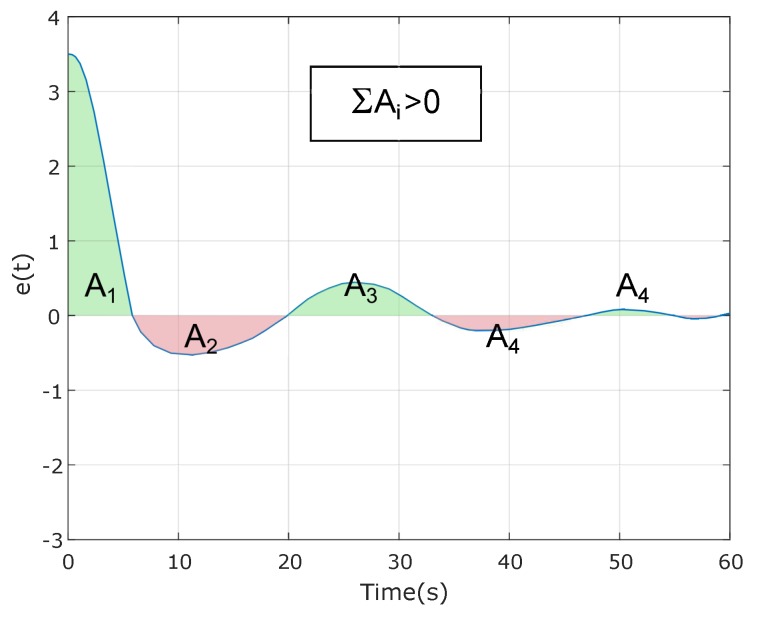
Error of a system formed by a reset controller with a double integrator plant.

To conclude whether the usage of reset control is convenient and advantageous over using linear regulation, a comparison must be established under fair conditions. This means that the reset controller must be compared with a linear one presenting good design characteristics. The problem is that finding a favorable linear regulator is not feasible because disturbance rejection imposes a maximum on slowness of the system, which is counterproductive for OS, acceleration and jerk. Therefore, getting a linear controller in compliance with all the design requirements is not possible since Equation (17) [39] holds for any linear controller similar in form as the one described in Equation (14) in a closed-loop with a double integrator plant, which fit the disturbance rejection specification. As a consequence, if the system conformed by the controller and the double integrator plant presents a positive or negative error, it will have to compensate for it by changing the error sign to reduce the cumulative term. Figure 16 shows graphically the implications of that limitation. Area $A_{1}$ is positive; thus, a second area with the opposite sign is required at least to decrease ∫e(t). Since $A_{2}$ is bigger than $A_{1}$, the error switches signs once again. These fluctuations continue until ∫e(t)=0. Since a reset system is not restricted by Equation (17) it can exhibit a time response as the one represented in Figure 17 where the sum of the areas is greater than zero:

(17) $$\begin{array}{c} \int_{0}^{\infty }e\left(t\right)dt=0. \end{array}$$

**Figure 18:**
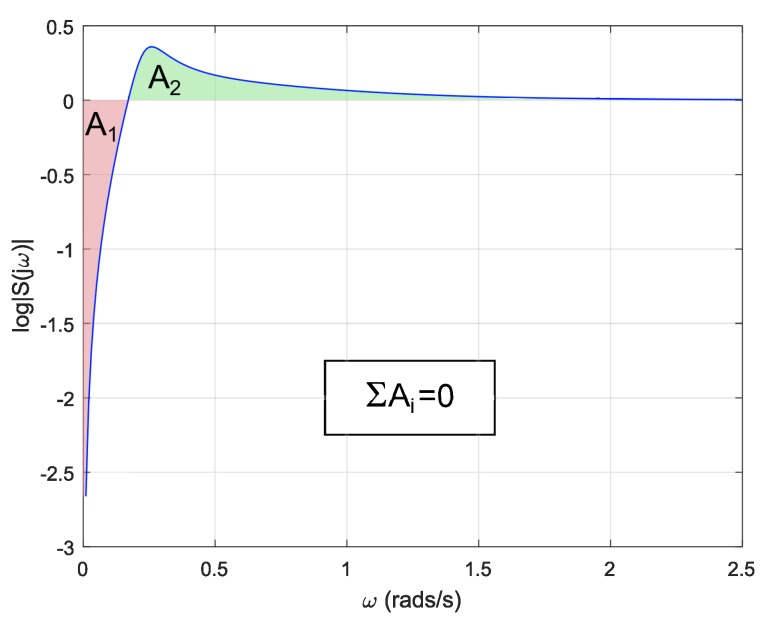
Sensitivity function of a linear system.

**Figure 19:**
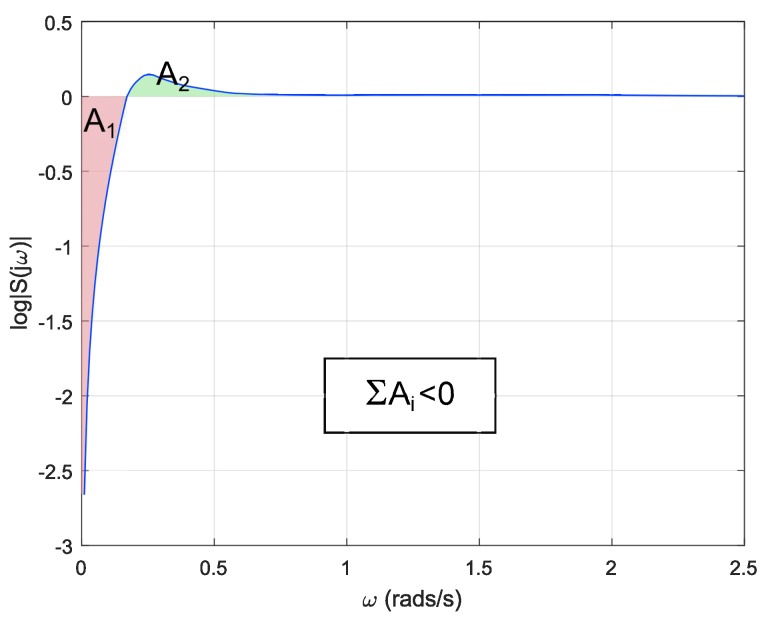
Sensitivity function of a reset system.

In addition to the previous limitation on the time domain, there are also restrictions on the frequency domain. Particularly, a linear system with a relative degree of its open-loop transfer function equal to or greater than two and none of its poles in the right-half plane, as it is the case for a double integrator plant, is subject to the following expression known as *Bode’s Integral Formula* [40]:

(18) $$\int_{0}^{\infty }\log\left|S\left(j\omega \right)\right|d\omega =0,$$

where S(jω) is the sensitivity function. This equation shows that, if sensitivity is suppressed at some frequency range, it is increased at some other range. This is known as the *waterbed effect*. Figure 18 shows a representation of the sensitivity function for different frequencies. As it can be seen, both colored areas must be equal to satisfy Equation (18). It must be noted that the upper area will equalize the lower one when *w* tend to infinity. Figure 19 shows the sensitivity function of a reset system. As it can be seen, the reset system is not restricted by Equation (18).

Since it is not possible to obtain a linear regulator that satisfies all the design criteria, all that can be expected is to fit the requirements separately. For instance, a controller with the following coefficients $a_{0}=0.0001$, $a_{1}=0.2006$, $a_{2}=0.8169$ and $a_{3}=1.2624$ produces a response with adequate values of jerk, acceleration, OS, $t_{s}$, $t_{r}$ but slow dynamics, leading to a poor robustness against disturbances. Due to the slow dynamics imposed by the controller, the system takes a large amount of time to take ∫e(t) to zero.

In contrast to a linear regulator, a system endowed with a reset mechanism is not subjected to the fundamental linear limitations. In a reset controller, it is possible to adjust the reset percentage and strategy to yield a lane change meeting all the design specifications. Three different reset strategies are compared, which are zero-crossing of the reset action, fixed-reset band and variable reset band. As far as reset percentage is concerned, full reset ($p_{r}=0$) is compared to an error-minimization method introduced in [27]. At every reset instant, the optimal $p_{r}$ in terms of ISE minimization is calculated, which is, in turn, based on $H_{2}$-norm minimization. For a function *E*∈R(s), the $H_{2}$-norm is defined as

(19) $${\left||E|\right|}_{2}=\sqrt{\frac{1}{2\pi }\int_{-\infty }^{\infty }{\left|E\left(j\omega \right)\right|}^{2}d\omega }.$$

Considering that, at any reset instant, the error is given by

(20) $$E_{k}\left(s\right)=C{\left(sI-A\right)}^{-1}x\left(t_{k}^{+}\right),$$

where $x\left(t_{k}^{+}\right)=\left(1-p_{r}\right)x\left(t_{k}\right)$, it can be concluded that its $H_{2}$-norm is

(21) $$\left|\right|E_{k}{\left|\right|}_{2}^{2}=x{\left(t_{k}^{+}\right)}^{⊤}Lx\left(t_{k}^{+}\right),$$

where *L* is the observability Gramian matrix, which is obtained from the following Lyapunov equation

(22) $$A^{⊤}L+LA+C^{⊤}C=0.$$

**Figure 20:**
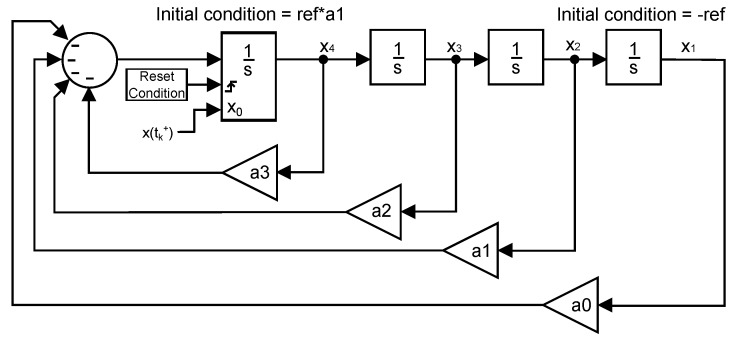
Canonical form of the autonomous system.

The previous Lyapunov equation has a unique solution if the eigenvalues $\alpha_{1},\alpha_{2},\ldots ,\alpha_{n}$ of $A^{⊤}$ and $\beta_{1},\beta_{2},\ldots ,\beta_{n}$ of *A* satisfy that $\alpha_{i}+\beta_{j}\neq 0$ for all pairs (i,j). If that condition is violated, there is not solution to the equation or it is not unique. For that reason, the system cannot use a non-autonomous realization to calculate the optimal pr for a step input since the resulting *A* matrix would have one pole at the origin caused by the step input. Consequently, an autonomous realization of the system is required. By adapting the canonical form described in Equation (12) and Figure 15, an autonomous equivalent system with Equations (23) and (24) can be easily obtained. Figure 20 shows a depiction of the system where no input is present:

(23) $$\left[\begin{array}{c} \dot{x_{1}}\\ \dot{x_{2}}\\ \dot{x_{3}}\\ \dot{x_{4}} \end{array}\right]=\left[\begin{array}{cccc} 0 & 1 & 0 & 0\\ 0 & 0 & 1 & 0\\ 0 & 0 & 0 & 1\\ -a_{0} & -a_{1} & -a_{2} & -a_{3} \end{array}\right]\left[\begin{array}{c} x_{1}\\ x_{2}\\ x_{3}\\ x_{4} \end{array}\right],$$

(24) $$y=\left[\begin{array}{cccc} 1 & 0 & 0 & 0 \end{array}\right]\left[\begin{array}{c} x_{1}\\ x_{2}\\ x_{3}\\ x_{4} \end{array}\right].$$

Once *L* is found and Equation (21) is also solved, the exact $p_{r}$, which minimizes the *ISE*, can be calculated. In order to calculate the optimal $p_{r}$ analytically, Equation (21) is rearranged as shown in Equation (25). Equation (26) can be readily obtained from the previous equivalences:

(25) $$\begin{array}{c} ISE=\left|\right|E_{k}{\left|\right|}_{2}^{2}=\left[\begin{array}{cccc} x_{1}\left(t_{k}^{+}\right) & x_{2}\left(t_{k}^{+}\right) & x_{3}\left(t_{k}^{+}\right) & x_{4}\left(t_{k}^{+}\right) \end{array}\right]\left[\begin{array}{cccc} L_{11} & L_{12} & L_{13} & L_{14}\\ L_{21} & L_{22} & L_{23} & L_{24}\\ L_{31} & L_{32} & L_{33} & L_{34}\\ L_{41} & L_{42} & L_{43} & L_{44} \end{array}\right]\left[\begin{array}{c} x_{1}\left(t_{k}^{+}\right)\\ x_{2}\left(t_{k}^{+}\right)\\ x_{3}\left(t_{k}^{+}\right)\\ x_{4}\left(t_{k}^{+}\right) \end{array}\right]\\ =\left[\begin{array}{cc} u^{⊤} & x_{4}\left(t_{k}^{+}\right) \end{array}\right]\left[\begin{array}{cc} L_{1} & L_{2}\\ L_{2}^{⊤} & L_{44} \end{array}\right]\left[\begin{array}{c} u\\ x_{4}\left(t_{k}^{+}\right) \end{array}\right], \end{array}$$

(26) $$ISE=u^{⊤}L_{1}u+2u^{⊤}L_{2}x_{4}\left(t_{k}^{+}\right)+L_{44}x_{4}^{2}\left(t_{k}^{+}\right)=\alpha x_{4}^{2}\left(t_{k}^{+}\right)+\beta x_{4}\left(t_{k}^{+}\right)+\gamma ,$$

where $\alpha =L_{44}$, $\beta =2u^{⊤}L_{2}$ and $\gamma =u^{⊤}L_{1}u$, all of them numbers. Therefore, as it can be seen, the integral quadratic error is equal to a function depending on $x_{4}$. The value of $x_{4}$ that minimizes the ISE is in Equation (27):

(27) $$x_{4}\left(t_{k}^{+}\right)=\frac{-\beta }{2\alpha }=\frac{-u^{⊤}L_{2}}{L_{44}}=\frac{x_{1}\left(t_{k}^{+}\right)L_{14}+x_{2}\left(t_{k}^{+}\right)L_{24}+x_{3}\left(t_{k}^{+}\right)L_{34}}{L_{44}}.$$

Replacing $x_{4}\left(t_{k}^{+}\right)=\left(1-p_{r}\right)x_{4}\left(t_{k}^{-}\right)$ and knowing that $x_{1}$, $x_{2}$ and $x_{3}$ are not reset leads to Equation (28) where the optimal $p_{r}$ can be seen:

(28) $$p_{r}=\frac{x_{1}\left(t_{k}^{-}\right)L_{14}+x_{2}\left(t_{k}^{-}\right)L_{24}+x_{3}\left(t_{k}^{-}\right)L_{34}+x_{4}\left(t_{k}^{-}\right)L_{44}}{x_{4}\left(t_{k}^{-}\right)L_{44}}.$$

The previous equation is computed at every consecutive reset instant resulting in an optimal $p_{r}$ sequence in terms of ISE. Combining this error minimization method with the canonical form guarantees the compliance of all the design requirements while reducing the error and enhancing the system output as a result. Equation (29) shows the computed value of *L* for a system formed by the controller introduced in Equation (16) and a double integrator plant:

(29) $$L=\left[\begin{array}{cccc} 6.2634 & 16.4957 & 16.5091 & 7.3234\\ 16.4957 & 100.0142 & 122.0803 & 64.1608\\ 16.5091 & 122.0803 & 153.1333 & 82.0887\\ 7.3234 & 64.1608 & 82.0887 & 44.6647 \end{array}\right].$$

Whenever the reset action is triggered, Equation (30) is used to calculate the optimal $p_{r}$:

(30) $$p_{r}=\frac{x_{1}\left(t_{k}^{-}\right)7.3234+x_{2}\left(t_{k}^{-}\right)64.1608+x_{3}\left(t_{k}^{-}\right)82.0887+x_{4}\left(t_{k}^{-}\right)44.6647}{x_{4}\left(t_{k}^{-}\right)44.6647},$$

where $x_{1}\left(t_{k}^{-}\right)$, $x_{2}\left(t_{k}^{-}\right)$, $x_{3}\left(t_{k}^{-}\right)$ and $x_{4}\left(t_{k}^{-}\right)$ are the lateral position and its time derivatives ($x_{1}=position$, $x_{2}=velocity$, $x_{3}=acceleration$ and $x_{4}=jerk$) just before the reset action is applied.

### 3.4. LQR and CNF Controllers

As stated in the introductory section, the designed reset controllers were compared with an LQR and a CNF regulator. The LQR works on the basis of a perfect pole-zero cancellation hence the double integrator plant was considered. Two other integrators are connected to the prefiltered plant to create a system with four states being the lateral position and its time derivatives ($x_{1}=position$, $x_{2}=velocity$, $x_{3}=acceleration$ and $x_{4}=jerk$). In this way, by applying Bryson’s law, the maximum values of the states can be enclosed to match the design specifications. The final arrangement of the system is equivalent to the one described in Figure 15 where $a_{0}$, $a_{1}$, $a_{2}$ and $a_{3}$ are now replaced by $k_{1}$, $k_{2}$, $k_{3}$ and $k_{4}$. Equation (31) defines the transfer function of the closed-loop system whose structure is identical to the system formed by the base linear controller and the double integrator plant:

(31) $$LQR_{CL}=\frac{k_{2}s+k_{1}}{s^{4}+k_{4}s^{3}+k_{3}s^{2}+k_{2}s+k_{1}}.$$

An LQR cannot produce a controller capable of meeting the design specifications all at once due to the fundamental limitations that affect linear systems. Therefore, the controller found by using this method (K=[0.00026 0.2619 0.8183 1.2793]) was adjusted to fulfill as many design requirements as possible. Acceleration, jerk, settling time, rise time and overshoot are restricted to their design ranges, whereas disturbance rejection exceeds the maximum allowed by far. This can be easily derived by computing the closed-loop poles and zeros. There is one small zero at $-k_{1}/k_{2}$ canceled by a small pole close to the origin. Since the LQR step response is restricted by the balance of error areas in Figure 16, the only way for an LQR to approach the step response of the reset control is to produce a certain type of slow hidden response, after the first zero-crossing, which is the main drawback of linear quadratic control.

Regarding the CNF regulator, it is based on the work developed in [31]. This paper investigated the use of CNF for the path following control problem for four-wheel independently actuated autonomous ground vehicles. Since the case study at issue differs from the one in [31], some adaptations had to be made. First, due to the fact that the control approach proposed in this work is conceived to be operational in a straight road section, the curvature term of Equation (9) in [31] is zero. Second, instead of considering two control signals as [31], this paper considers only δ.

**Figure 21:**
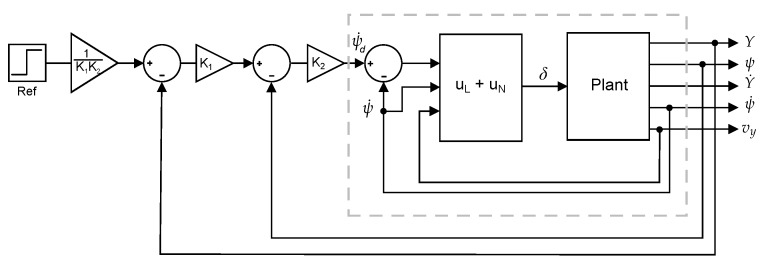
Schematic depiction of the CNF controller.

Figure 21 contains a schematic depiction of the CNF regulator coupled with the plant of the vehicle. There are two distinguishable zones. A block marked as $u_{L}+u_{N}$ which will be referred, hereinafter, as the internal loop and the rest of the blocks, excluding the plant, which will be referred as the external loop.

As mentioned in Section 1, CNF is a composite nonlinear control technique consisting of a linear and a nonlinear control law directly connected. Both the lineal and the nonlinear feedback laws are represented in Equations (32) and (33), respectively:

(32) $$u_{L}=F\left[\begin{array}{c} \psi \\ v_{y} \end{array}\right]+Gr+G_{r}\dot{r},$$

(33) $$u_{N}=\rho \left(r,x\right)B^{⊤}P\left[\left[\begin{array}{c} \psi \\ v_{y} \end{array}\right]-\bar{G}r\right],$$

where *F*, *G*, $G_{r}$, ρ and *P* are defined in [31]. *r* is equal to ${\dot{\psi }}_{d}$, this being the desired yaw rate and it is equal to $-k_{2}\left(\psi +k_{1}y\right)$. $v_{y}$ is not equivalent to $\frac{dy}{dt}$, which is, however, equal to $v_{x}\sin\left(\psi \right)+v_{y}\cos\left(\psi \right)$. To simplify the comparison and facilitate the design, it is assumed that $v_{y}$ is measured and $G_{r}=0$.

The total control actuation signal is the result of adding the linear and the nonlinear parts as showed in Equation (34). This control law is particularized for a vehicle traveling at a longitudinal velocity of 25 m/s and without extra loads:

(34) $$u=-1.6295\psi +0.6050v_{y}+2.0699{\dot{\psi }}_{d}-3e^{-100|{\dot{\psi }}_{d}-\psi |}\left[\begin{array}{cc} 0.5923 & 0.7235 \end{array}\right]\left[\left[\begin{array}{c} \psi \\ v_{y} \end{array}\right]-\left[\begin{array}{c} 1\\ -0.4247 \end{array}\right]{\dot{\psi }}_{d}\right].$$

**Figure 22:**
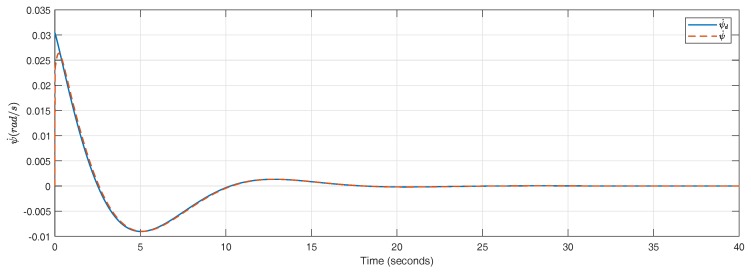
${\dot{\psi }}_{d}$ and $\dot{\psi }$.

As it can be seen in Figure 22, the yaw rate is perfectly tracked. Since the internal loop ($u=u_{L}+u_{N}$) presents such fast dynamics, the whole system depicted in Figure 21 can be approximated by the open-loop transfer function in Equation (35):

(35) $$CNF_{OL}=\frac{k_{1}k_{2}\left(v_{x}+cs\right)}{s^{2}+k_{2}s},$$

where $v_{x}$ is the longitudinal velocity of the vehicle and *c* can be obtained from the following equation:

(36) $$\left[\begin{array}{c} 1\\ c \end{array}\right]=-{\left(A+BF\right)}^{-1}BG.$$

From the previous transfer function, $k_{1}$ and $k_{2}$ can be readily tuned to obtain a convenient response in an approximate manner ($k_{1}=0.0175$ and $k_{2}=0.4968$). The complete system is simulated to test the performance of both internal and external loops connected together. The result of this simulation presents some issues concerning acceleration and jerk at the beginning of the maneuver. As soon as the vehicle initiates the maneuver, the acceleration produced by the control system is steep enough to produce unbounded jerk.

Acceleration and jerk can be restricted to its optimal range, in terms of comfort by filtering the error signal to attenuate them at the beginning of the maneuver. From Equation (35), this filtering would be achieved by means of using an intermediate first-order filter that is represented in Equation (37) by $\frac{k_{3}}{S+k_{3}}$. This method has some disadvantages regarding the dynamics of the resulting system. Ideally, $k_{3}$ should be chosen to filter the initial peaks and to have an effect as little as possible on the CNF setup. In order for the filter to accommodate jerk to the comfort zone, its value would have to be so small that the resulting system would have a slow pole producing, as a result, high values of settling and rise time. $k_{3}$ was finally selected at 0.35 as an intermediate solution. Even though it is true that the resulting system does not meet the comfort requirements, it at least produces bounded values of jerk:

(37) $$CNF=\frac{k_{3}}{s+k_{3}}\frac{k_{1}k_{2}\left(v_{x}+cs\right)}{s^{2}+k_{2}s}.$$

### 3.5. Simulation Results

This section is dedicated to the comparison of all the reset strategies mentioned in the previous section as well as two other controllers based on linear-quadratic control and composite nonlinear feedback control. For the following simulations, a perfect pole-zero cancellation is assumed and, therefore, neither prefilters nor complete plants are used but the double integrator. This prerequisite holds for all the reset controllers and the linear-quadratic regulator. The CNF regulator, however, was designed for the complete model so the system is not guaranteed to operate correctly for any conditions differing from those taking into account in the design of the controller.

**Figure 23:**
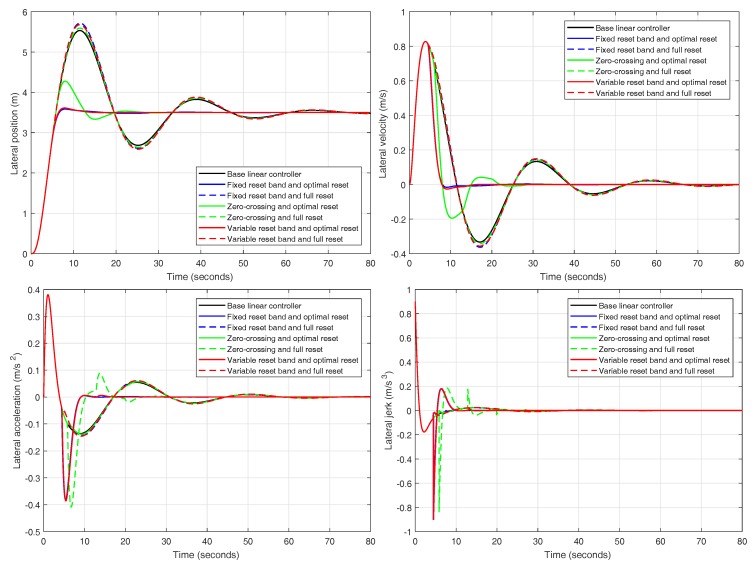
Position, velocity, acceleration and jerk for different reset controllers in simulation.

Firstly, results from the reset controllers are compared separately to determine which of the reset strategies present better responses. Figure 23 shows the lateral position, velocity, acceleration and jerk of the lane change maneuver for each of the six reset regulators compared together with the base linear controller. For the fixed reset band controllers, the band is set to 0.31 and, for the variable reset band regulators, the band is set to 1.27. Both values were selected by design convenience based on simulation results.

In view of the information collected in Table 4 and the results depicted in Figure 23, it can be firmly concluded that those controllers that employ the ISE minimization method outperform those endowed with a full reset action, regardless of the reset strategy. Among the regulators using the minimization technique, it is difficult to discern which one is the best since two of them present very similar responses (fixed and variable reset band controllers).

**Figure 24:**
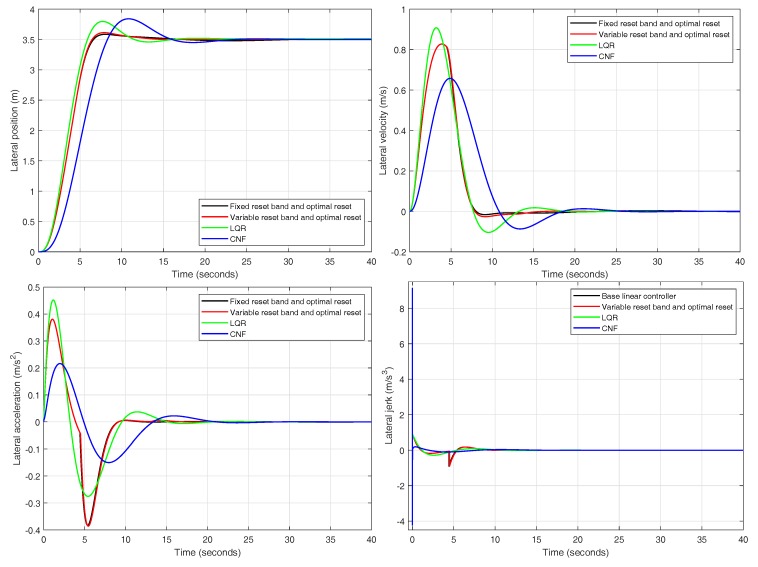
Position, velocity, acceleration and jerk for the following controllers: variable reset band and optimal reset, fixed reset band and optimal reset, linear-quadratic and CNF.

Two of the reset controllers meet all the design criteria so only them are compared together with the linear-quadratic and the CNF regulators in Figure 24. As stated in Section 3.4, none of the two alternative controllers fully satisfy all the design specifications. The linear-quadratic regulator lacks an adequate disturbance rejection ratio surpassing by far the design criterion set at $max\left|\frac{Y_{D}}{D}\right|=0.005$. The main problem with this controller is the hidden slow dynamics commented in Section 3.4. The main reason for this problem is that LQR (as any other linear control) is affected by the error area restriction in Figure 16, which only nonlinear controllers are able to overcome. The CNF controller exhibits good performance, but, in the form presented in [31], it is restricted to the internal loop (yaw rate control), which has an excellent performance, as seen in Figure 22. The solution proposed in [31] for the external loop, based on gains $k_{1}$ and $k_{2}$, is purely linear and does not exploit the full potentials on CNF. Another advantage of [31] is the use of a nested cascade structure that takes full advantage of gyroscope yaw rate measurements. Future work could consider an extension of [31] that also includes CNF in the external loop.

**Figure 25:**
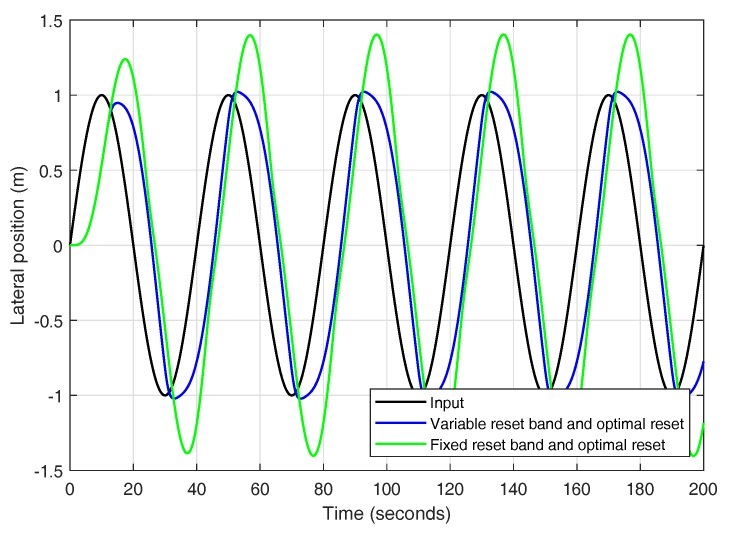
Variable input.

Even though this work focuses on a lane change from a control perspective at low level, it may be of interest to test the responses of the reset controllers for a variable input just as it would be in a more realistic scenario. Only those reset strategies meeting all the design criteria are tested for a variable input. In this case, a sine wave of period 40 s is chosen as the input. In Figure 25, it can be seen how a variable reset band controller can adapt better to a varying input as opposed to a fixed reset band controller that is designed to work with a particular input reference.

**Figure 26:**
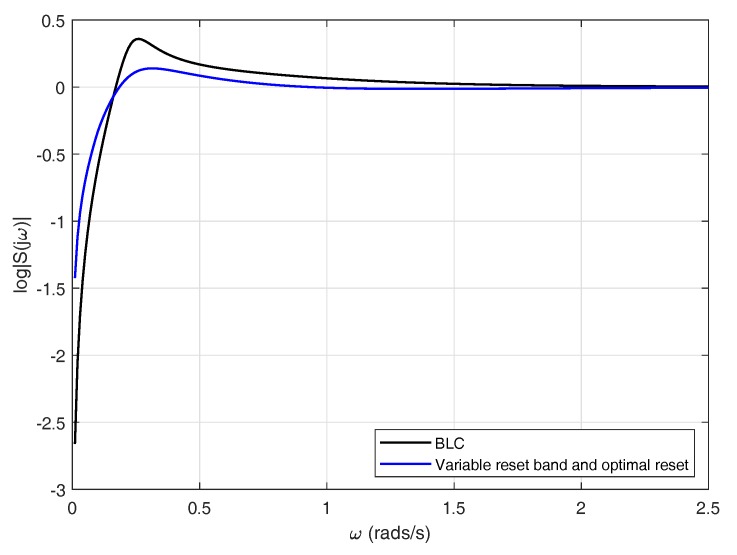
Sensitivity functions.

As mentioned previously, in addition to constraints on the time domain, linear systems also exhibit restrictions on the frequency domain. Endowing a linear controller with a reset strategy may redound to improvements on the frequency response of the system. It can be concluded from Figure 26 that using a variable reset band with optimal reset yields a controller with an enhanced frequency response. The base linear controller presents a maximum around 0.26 rad/s, whereas the reset controller attenuates that peak. Computing the sum of the areas of the estimated sensitivity function in the reset case produces a value different from zero −0.0550, the positive area being equivalent to 34.42% of the negative area. This is due to the fact that the BLC is restricted by Equation (18) while the reset controller is not, for this reason a suppression of the sensitivity function at some frequency range does not necessarily imply an increment in other frequencies.

## 4. Validation

In this section, the experiments described in Section 3.5 are performed with the help of CarSim Simulation Software. A closed-loop system is proposed based on the system depicted in Figure 14, but, instead of using a double integrator, this plant is replaced by CarSim.

The system is tested for a longitudinal speed of 25 m/s and using the prefilter represented in Table 3. The control arrangement selected is based on the canonical system shown in Figure 20 and controller (16) is used. As it was mentioned before, this canonical form is selected because the jerk of the vehicle can be limited.

**Figure 27:**
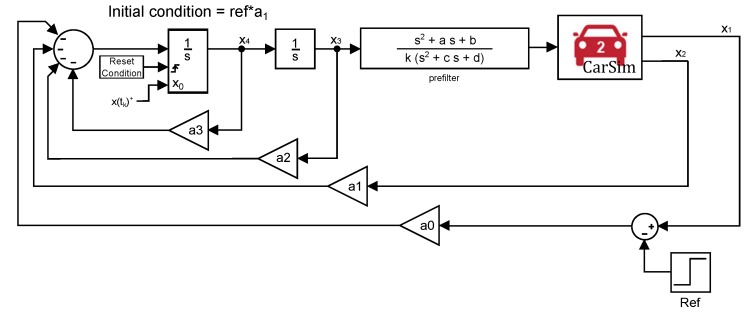
System used for validation.

The system indicated in Figure 27 results from combining controller, prefilter and real plant (CarSim) as shown in the control scheme of Figure 20. This is the setup used to perform the experiments in validation. It must be taken into account that the real car, CarSim, is not perfectly prefiltered as it occurs in the theoretical approach, Figure 14 of Section 3.2. There exist uncertainties and differences between model and real car and, therefore, the plant is slightly different to a double integrator.

The states of the system are $x_{1}$ = position, $x_{2}$ = velocity, $x_{3}$ = acceleration, $x_{4}$ = jerk. As it can be seen, the states $x_{1}$ and $x_{2}$ are taken from CarSim software directly instead of getting them from integrator blocks. The states $x_{3}$ and $x_{4}$ are part of the controller, being this last state, $x_{4}$, the selected state to be reset to a different value every time the reset condition is reached, as it was explained in Section 3.1.

### 4.1. Comparison of the Control Strategies

**Figure 28:**
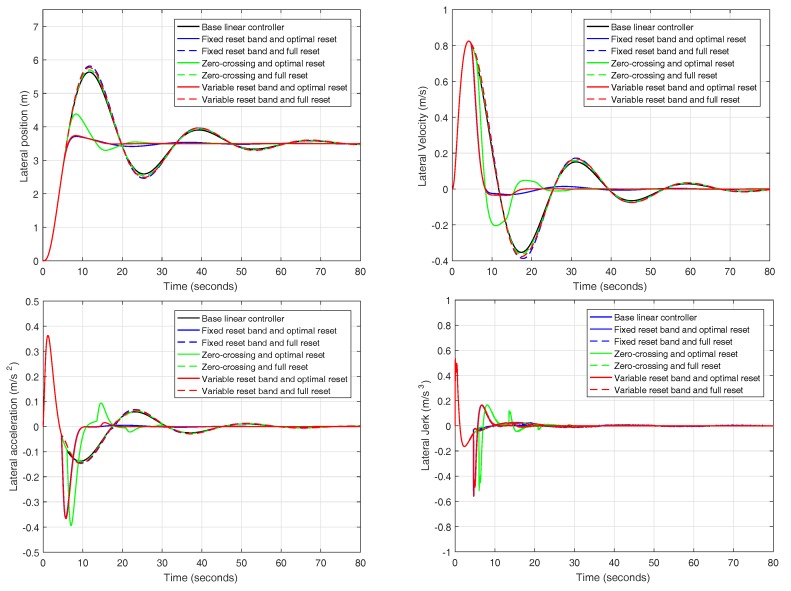
Position, velocity, acceleration and jerk for different reset strategies in CarSim.

Firstly, a comparison of all the reset control strategies studied in Section 3 is presented. As it can be observed in Figure 28 and Table 5, the response of the car is very similar to the results presented in Section 3.5 for the different experiments. Some differences exist between the response of the system obtained with CarSim and the response obtained in simulation (Figure 23) because a real plant with a prefilter is introduced. Anyway, the controller designed for a double integrator plant behaves correctly in CarSim, obtaining a reasonable response.

The controller must satisfy high performance specifications such as short rise time, short settling time, low values of acceleration and jerk in lateral displacement and low overshoot. Due to these restrictive specifications, it was concluded that the best controller is the one endowed with a *variable band and optimal reset percentage*, which meets all the specifications required by design.

**Figure 29:**
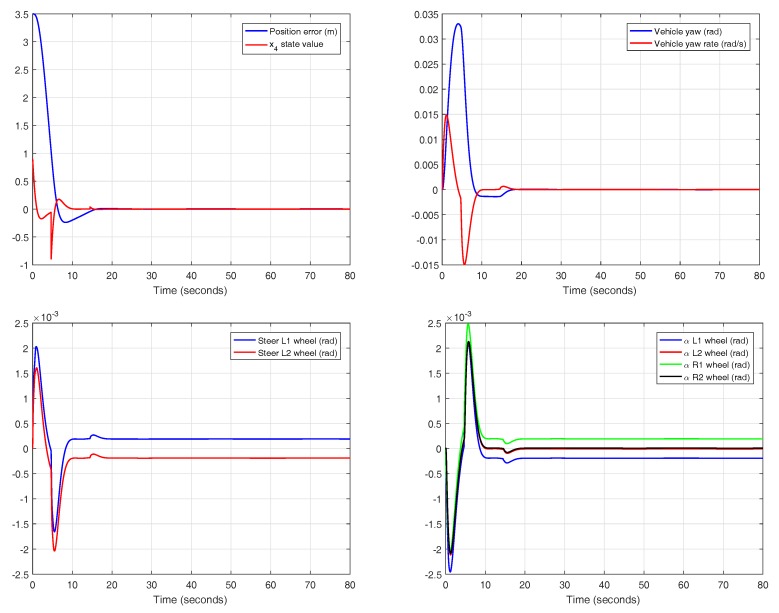
Detail of the experiment for the controller with variable band and optimal reset in CarSim.

Selecting the controller with optimal reset and variable band, other variables of the system can be shown, as seen in Figure 29. In this case, the reset action is applied when the condition of the variable band, with h=1.27, is reached. The state $x_{4}$ of the controller is reset, satisfying the jerk limit of 0.9 m/s${}^{3}$. It must be noted that the real jerk of the vehicle is slightly different to the $x_{4}$ state of the controller, contrary to what happens in the theoretical approach.

The system input is the steering angle of the front wheels. In this case, the reset action does not require excessive effort in the actuator, keeping the values in a low range. Since the slip angles are also restricted to small absolute values, the assumptions made in the theoretical approach are confirmed.

As it was said in Section 3.2, a linear controller cannot satisfy all the design requirements at the same time. If the controller is designed to be robust against disturbances, the requirements of low acceleration and jerk cannot be satisfied. In addition, if it is designed with respect to the comfort limits, it can satisfy other requirements but not all of them. Reset control allows for designing a robust system at the same time that high performance as well as low overshoot is achieved.

The controller with variable band and optimal reset is used in the next experiments where the controller will be tested against parametric uncertainties and external disturbances.

### 4.2. Validation of the Prefilters for Changing Longitudinal Velocity

Although the previous experiments were performed for a constant longitudinal velocity, the maneuver can be done with a varying longitudinal speed. As the prefilter depends on the velocity of the vehicle, this must be exchanged for each longitudinal velocity range. The transfer functions employed are shown in Table 3, which have been calculated beforehand for the different speed ranges considered.

**Figure 30:**
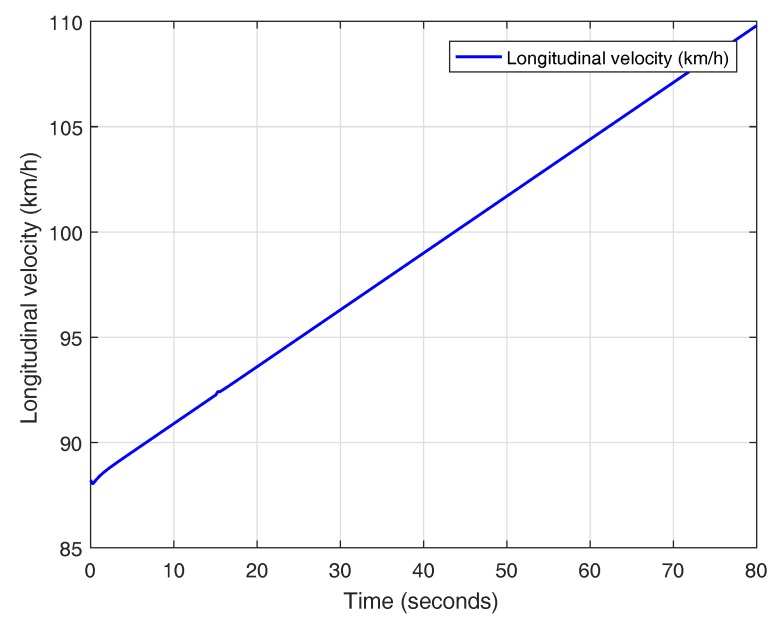
Variation of the longitudinal velocity in Carsim.

**Figure 31:**
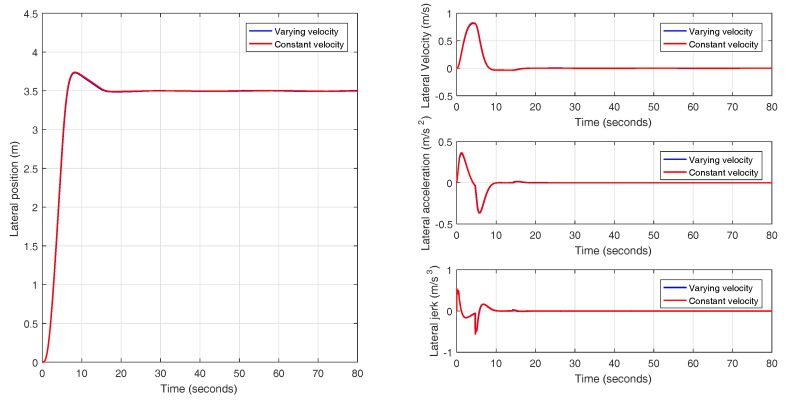
Validation of the controller with variable band and optimal reset for varying longitudinal velocity (88.2–109.8 km/h) in CarSim.

In this experiment, a lane change maneuver is performed with an initial speed of 88.2 km/h (24.5 m/s) with a uniform increase of velocity to a final value of 109.8 km/h (30.5 m/s), as it is shown in Figure 30. The results obtained for the lane change maneuver with varying velocity are depicted in Figure 31. Based on what has been observed, it can be concluded that switching the prefilter does not affect the performance of the system and the prefiltered plant behaves as a double integrator, as it was explained in Section 3.2.

### 4.3. Response of the System for Parametric Uncertainties in the Model

The real car can present some differences with the identified model. The parametric uncertainties inherent to the identification process or other kind of uncertainties may exist, such as changes in the load of the car or its distribution. This section shows how the car behaves when it is loaded with more weight, in particular, five passengers, as it was described in Section 2.3.

**Figure 32:**
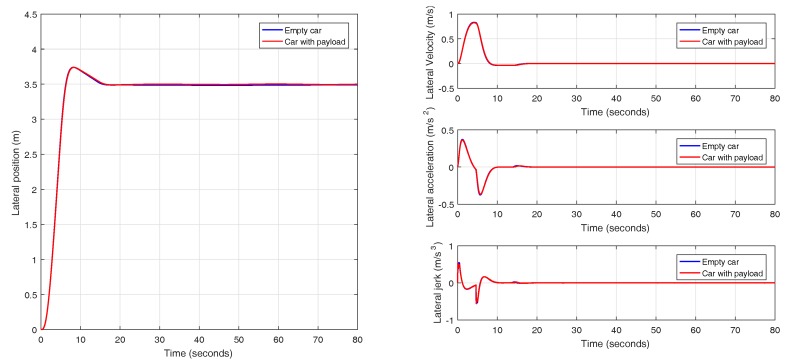
Validation of the controller with variable band and optimal reset with payload at 90 km/h in CarSim.

**Figure 33:**
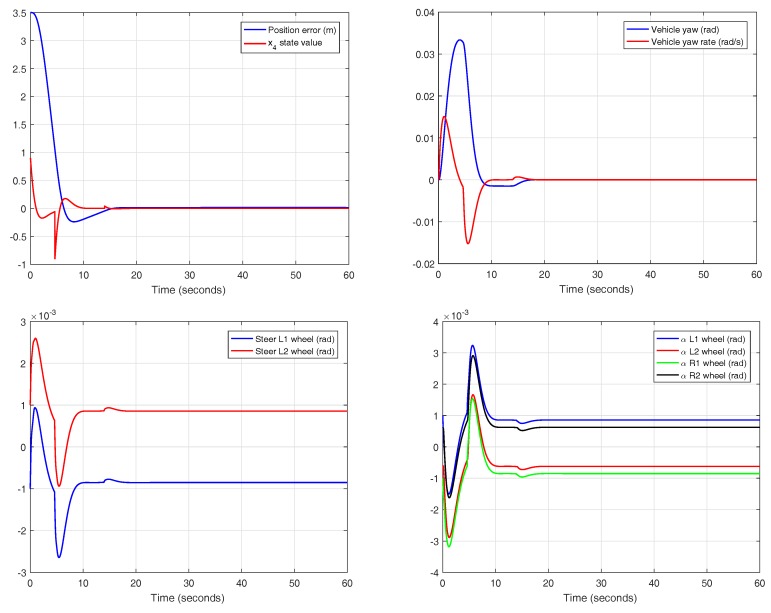
Detail of the experiments for the controller with variable band and optimal reset with payload at 90 km/h in CarSim.

In this case, a car traveling at a speed of 90 km/h is selected and the passengers are distributed as it is shown in Figure 8. In Figure 32, the comparison between the response (position, speed, acceleration and jerk) of an empty and a loaded vehicle can be seen. Figure 33 shows other parameters. The response of the loaded car is very similar to the response of the empty car, and this is mainly due to the preciseness of the calculated prefilter, which reduces the influence of variability in the model parameters.

### 4.4. Response of the System to External Disturbances

In the next experiment, the effect on the vehicle of an external force is analyzed for the maneuver under study. As mentioned above, although the main functionality of the controller is to provide a swift and smooth lane change, the system must exhibit a certain degree of disturbance rejection for the sake of safety. A lateral force representing the wind, which induces a deviation in the car position, can be set in CarSim.

The controller was selected to produce small gains for the transfer function in Equation (7), as described in Section 3.3. In the real system, the disturbance rejection gain is worse than the one obtained with the theoretical approach because of the differences between model and real plant. Then, it is necessary to test the real plant and specify the maximum value of overshoot in the maneuver that the controller can handle.

**Figure 34:**
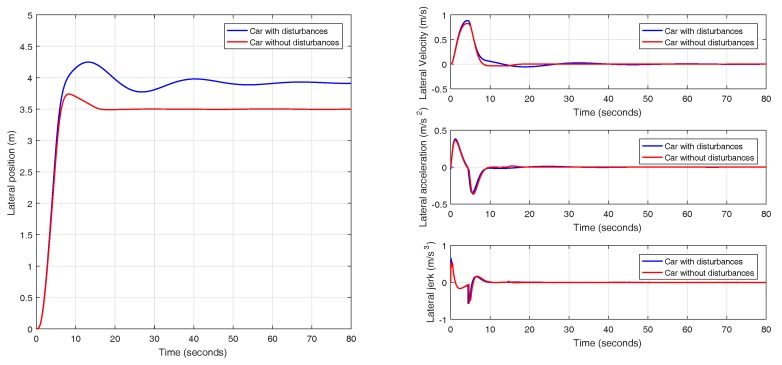
Validation of the controller with variable band and optimal reset with an external disturbance in CarSim.

**Figure 35:**
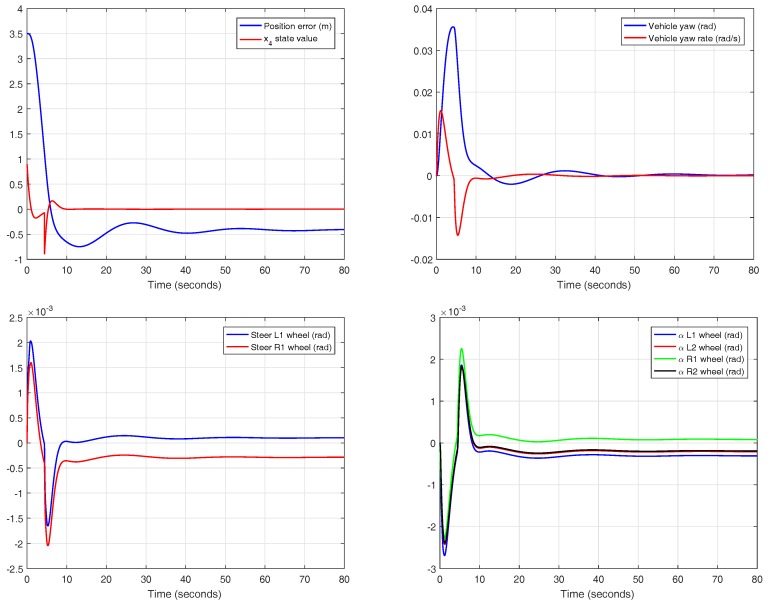
Detail of the experiment for the controller with variable band and optimal reset with an external disturbance in CarSim.

**Figure 36:**
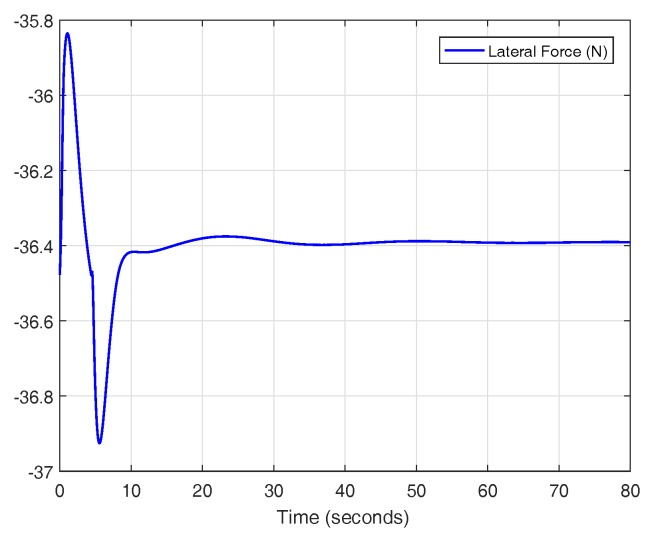
Wind force on the vehicle for the controller with variable band and optimal reset in CarSim.

The experiment is performed by establishing a wind force value on the right side of the vehicle, where lane departure is more dangerous. The results of the experiment are shown in Figure 34 and Figure 35. For a controller with a variable reset band and optimal reset, the system has an overshoot of 0.24 m without any disturbances (6.8%). Since the trajectory of the car has to be inside of the lane width (4.25 m), the vehicle has 0.51 m left within the lane. In this case, the average force value admitted by the controller is 36.4 N (see Figure 36). Thus, the gain of Equation (7), with a value of 0.014 m/N, is not as good as the gain of the theoretical model. In any case, this is not considered a problem because, as mentioned before, the compensator focuses on changing lane and it could be replaced by a lane keeping controller as soon as the maneuver has been completed.

## 5. Conclusions

In this work, various reset controllers were studied for a lane change maneuver under a set of restrictive design specifications selected with the objective of ensuring ride quality at all times as well as a swift response. On account of the comfort requirements, it was necessary to rearrange the dynamical model employed to limit the jerk signal. To get to this realization, a prefiltering method was conceived to homogenize the resulting system and make it independent of the maneuver conditions with the exception of the longitudinal velocity, which is employed to adjust its design. Every prefilter is restricted to an operational range of velocities where it is guaranteed to yield an accurate pole-zero cancellation. This method could be used to readily extend its effectiveness over a wider range of velocities by obtaining more prefilters. This way, the system would have to consult the corresponding prefilter for each velocity in a lookup table. For the sake of simplicity and exemplification, a limited group of prefilters was included in the paper.

The base linear controller was conveniently obtained via genetic algorithms for the system resulting from combining the prefilter and the vehicle model, i.e., the double integrator plant. The use of this optimization method did not produce any linear controller capable of satisfying all the design requirement simultaneously.

Additionally, to increase confidence in the feasibility and applicability of the method, those reset strategies that were selected to be the best in terms of design specifications and performance were also compared with an LQR and a CNF controller. The linear-quadratic approach does present the same fundamental limitations that the base linear controller so that meeting all the design requirements all at once is not possible. Concerning the CNF control, while it is true that it presents some advantageous characteristics, such as a perfect tracking of a varying yaw rate or a good transient performance, due to the demanding scenario considered, this method had to be discarded.

Finally, it could be concluded that, by combining the calculation of $p_{r}$ by means of a Lyapunov-based ISE minimization method and the use of a variable reset band together with the limitation of the jerk signal, all the design specifications could be met. This was supported by the simulations performed with CarSim, based on a high-fidelity virtual vehicle that includes all real nonlinearities, which included the influence of parametric uncertainty, changing velocity and the effect of external disturbances on the system. The previous simulations also demonstrated the accuracy of the small-angle assumption considered for the linear models during the design part.

## Figures and Tables

**Table 1:** Parametric variation of a Sedan-D 2017.

Parameter	Description	Empty Car	Loaded Car	Units
$l_{f}$	Distance from the front axle to C.G.	1.11	1.25	m
$l_{r}$	Distance from the rear axle to C.G.	1.67	1.53	m
*M*	Mass of the vehicle	1370	1770	kg
$I_{z}$	Momentum of Inertia of the vehicle	2315	2535	kg m${}^{2}$

**Table 2:** Variation of the model parameters.

Parameter	Minimum Value	Maximum Value	Units
$l_{f}$	1.11	1.25	m
$l_{r}$	1.53	1.67	m
*M*	1370	1770	kg
$I_{z}$	2315	2535	kg m${}^{2}$
$C_{f}$, $C_{r}$	88,000	112,000	N/rad

**Table 3:** Prefilters.

Interval of Velocities	Prefilter
[24.5,25.5)	$0.0078272\frac{s^{2}+23.27s+164.5}{s^{2}+14.68s+228.9}$
[25.5,26.5)	$0.0078364\frac{s^{2}+22.38s+154.7}{s^{2}+14.13s+229}$
[26.5,27.5)	$0.0078278\frac{s^{2}+21.54s+145.6}{s^{2}+13.57s+228.6}$
[27.5,28.5)	$0.007813\frac{s^{2}+20.81s+138.5}{s^{2}+13.12s+229}$
[28.5,29.5)	$0.0077736\frac{s^{2}+20.16s+132.3}{s^{2}+12.7s+229.5}$
[29.5,30.5)	$0.0077933\frac{s^{2}+19.43s+125.2}{s^{2}+12.23s+228.8}$

**Table 4:** Simulation characteristics of the controllers.

Controller	ISE	∫*e(t)*	$t_{r}$ (s)	$t_{s2\%}$ (s)	0S (%)
Base linear controller	66.768	0	3.704	57.365	58.088
Zero-crossing and full reset	69.169	−0.274	3.704	57.937	59.793
Fixed reset band and full reset	73.071	−1.213	3.697	57.721	63.309
Variable reset band and full reset	72.248	−0.711	3.699	58.002	62.191
Zero-crossing and optimal reset	35.902	9.786	3.703	17.975	22.215
Fixed reset band and optimal reset	34.009	12.257	3.844	9.266	2.425
Variable reset band and optimal reset	34.003	12.097	3.814	9.866	3.208
Linear-quadratic regulator	31.913	0	3.582	10.389	8.157
Composite nonlinear feedback controller	47.595	15.766	4.911	14.825	9.721

**Table 5:** Validation characteristics of the controllers.

Controller	ISE	∫e(t)	$t_{r}$ (s)	$t_{s2\%}$ (s)	0S (%)
Base linear controller	73.185	−0.400	3.704	72.509	62.234
Zero-crossing and full reset	76.041	−0.724	3.699	73.465	64.290
Fixed reset band and full reset	80.814	−1.294	3.695	73.105	67.679
Variable reset band and full reset	79.151	−1.105	3.691	73.708	66.753
Zero-crossing and optimal reset	38.598	9.823	3.725	19.117	25.397
Fixed reset band and optimal reset	36.130	12.452	3.808	24.452	6.340
Variable reset band and optimal reset	36.087	11.964	3.799	13.749	6.866
